# Amphiphilic Polypeptides Obtained by the Post-Polymerization Modification of Poly(Glutamic Acid) and Their Evaluation as Delivery Systems for Hydrophobic Drugs

**DOI:** 10.3390/ijms24021049

**Published:** 2023-01-05

**Authors:** Apollinariia Yu. Dzhuzha, Irina I. Tarasenko, Leonard Ionut Atanase, Antonina Lavrentieva, Evgenia G. Korzhikova-Vlakh

**Affiliations:** 1 Institute of Chemistry, Saint-Petersburg State University, 198504 St. Petersburg, Russia; 2 Institute of Macromolecular Compounds, Russian Academy of Sciences, 199004 St. Petersburg, Russia; 3 Faculty of Dental Medicine, “Apollonia” University, 700399 Iasi, Romania; 4 Institute of Technical Chemistry, Gottfried-Wilhelm-Leibniz University, 30167 Hannover, Germany

**Keywords:** post-polymerization modification, amphiphilic poly(amino acids), polypeptides, nanoparticles, drug delivery, hydrophobic drugs, paclitaxel

## Abstract

Synthetic poly(amino acids) are a unique class of macromolecules imitating natural polypeptides and are widely considered as carriers for drug and gene delivery. In this work, we synthesized, characterized and studied the properties of amphiphilic copolymers obtained by the post-polymerization modification of poly(α,L-glutamic acid) with various hydrophobic and basic L-amino acids and D-glucosamine. The resulting glycopolypeptides were capable of forming nanoparticles that exhibited reduced macrophage uptake and were non-toxic to human lung epithelial cells (BEAS-2B). Moreover, the developed nanoparticles were suitable for loading hydrophobic cargo. In particular, paclitaxel nanoformulations had a size of 170–330 nm and demonstrated a high cytostatic efficacy against human lung adenocarcinoma (A549). In general, the obtained nanoparticles were comparable in terms of their characteristics and properties to those based on amphiphilic (glyco)polypeptides obtained by copolymerization methods.

## 1. Introduction

The design and development of new biocompatible and biodegradable polymers for biomedical applications is one of the most dynamic areas of modern science. Bio-inspired and biomimetic (nano)materials are of particular interest due to their biodegradability to non-toxic natural metabolites and their ability to provide an additional biological functionality. Synthetic poly(amino acids) represent a unique class of macromolecules imitating natural polypeptides, which are widely considered as carriers for drug and gene delivery [1,2,3] as well as vaccines development [4].

The main approach to synthesizing poly(amino acids) with a non-predetermined primary structure is the ring opening polymerization (ROP) of N-carboxyanhydrides (NCAs) of α-amino acids or their derivatives [5]. Depending on the nature of the initiator, ROP NCAs can provide (co)polymers with different molecular weights and dispersity [6,7,8,9,10,11]. An alternative way to obtain polypeptides bearing the functional moieties of interest is the post-polymerization modification of a poly(amino acid) main chain [12]. In the case of poly(amino acids), this approach is mainly used to modify them with thermo-responsive moieties [12], PEG [12], sugars [8,13] and hydrophobic molecules [14,15]. In particular, Xue et al. reported the modification of poly(L-ornithine-*co*-L-citrulline) with N-succinimidyl N-methylcarbamate to obtain thermo-responsive copolymers. Post-modification with sugars allows for the ease of the preparation of glycopolypeptides that mimic natural macromolecules and can be utilized for processes based on molecular recognition. Isothiocyanate functionalized mannose [16], D-gluconolactone [17], fructose [18], galactose and glucose [19] as well as D-glucosamine [13,20] are utilized to modify poly(L-lysine) [8,16,17,18] and poly(L-glutamic acid) [13,20]. Amidation and azide-alkyne cycloaddition are among the most popular modification techniques [8,21]. In the case of hydrophobization, the modifications of poly(glutamic acid) with 4-phenylbutanol [15], *p*-aminoazobenzene [22], Boc-protected α,ω-aminoalcohols containing different alkyl spacers [23], cinnamyl alcohol [24] and coumarin derivative [25] are reported. The modification of poly(L-lysine) with L-histidine is one of the most popular approaches to obtaining polypeptides sensitive to physiological pH values [26,27,28].

Due to their biocompatibility and biodegradability to non-toxic metabolites, the amphiphilic polypeptides are very promising candidates for the delivery of various drug [29,30,31,32,33]. There are a number of works devoted to the development of the polypeptide-based conjugates and nanoparticles as delivery systems for peptides [34,35], nucleic acids [36,37], cytostatic drugs [38,39,40] and some others [41]. Furthermore, a pharmaceutical formulation of the anti-tumor drug based on poly(glutamic acid) (P[E]) and produced by Cell Therapeutics (Souse Korea) is commercially available under the tradename Poliglumex^®^ (conjugate of P[E] with paclitaxel, formerly Xyotax^®^) [42].

In this study, we aimed at the synthesis of amphiphilic (glyco)polypeptides by the post-polymerization modification of poly(glutamic acid), the formation and characterization of nanoparticles from the polymers obtained as well as their in vitro biological evaluation. Given that nanoparticles formed from the amphiphilic nanoparticles are known to be suitable for the effective encapsulation of hydrophobic drugs [43], the synthesized amphiphilic (glyco)polypeptides were examined for their potential applicability as delivery systems for paclitaxel (PTX).

PTX is one of the very effective cytostatic drugs in the first-line cancer treatment, especially in the cases of breast and ovarian cancer and a wide spectrum of carcinomas of the lung, prostate and brain [44,45]. A significant disadvantage of paclitaxel is its hydrophobicity and insolubility in aqueous media, and, as a result, its low bioavailability. In this regard, it is considered that nanoformulated PTX can significantly improve patient outcomes in cancer chemotherapy [46,47,48].

Here, the developed nanoparticles were characterized for their size and surface charge, morphology, storage stability, cytotoxicity to normal lung cells and ability to uptake by macrophages. The encapsulated forms of PTX based on the developed nanoparticles were obtained, and the in vitro cytostatic activity of PTX nanoformulation was evaluated using human lung adenocarcinoma cells (A549 cell line). The results obtained were discussed in comparison with previously published data for nanoparticles and their PTX formulations based on amphiphilic (glyco)polypeptides synthesized by the ROP.

## 2. Results and Discussion

### 2.1. Synthesis of Poly(L-Glutamic Acid) and Its Post-Polymerization Modification

The possibility of forming nanoparticles based on biocompatible poly(L-glutamic acid) modified with various amino acids and N-glucosamine was studied in this work. As hydrophobic amino acids responsible for nanoparticle self-assembly and the capture of hydrophobic drugs, L-phenylalanine, L-isoleucine and L-tryptophan were selected. The basic L-ornithine and L-arginine were used to provide intraparticle electrostatic interaction with carboxylic groups of glutamic acid and additionally stabilize the delivery systems. Saccharide units were introduced to provide additional colloidal stability in the model and physiological media.

**Figure 1:**
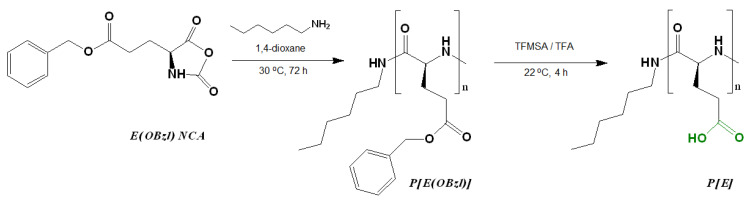
Scheme of the synthesis of poly(α-glutamic acid) by the ring-opening polymerization of the N-carboxyanhydride of L-glutamic acid γ-benzyl ester.

The synthesis of poly(L-glutamic acid) (P[E]) was carried out by a two-step procedure which included (1) the ROP of the L-glutamic acid γ-benzyl ester NCA initiated with *n*-hexylamine and (2) the deprotection of the resulting hydrophobic copolymer by the acidic hydrolysis of the benzyl ester (Figure 1). Before deprotection, the intermediate P[E(OBzl)] was analyzed by size-exclusion chromatography (SEC) in DMF (Figure S1, Supplementary Materials). According to SEC, the average molecular weight (*M_n_*) and dispersity (*Ɖ*) of P[E(OBzl)] were 14,400 and 1.40, respectively. In addition, the *M_n_* of P[E(OBzl)] was calculated by ^1^H NMR spectroscopy (Figure S2, Supplementary Materials) and was equal to 10,000. The difference in the *M_n_* values determined by SEC and NMR can be explained by the fact that the used SEC mode included molecular weight calculations relative to poly(methyl methacrylate) standards.

After deprotection, a 94% removal of Bzl-groups was confirmed by the significant reduction in the signals at 7.04 ppm (-CH_2_C_6_H_5_) and 5.28 ppm (-CH_2_C_6_H_5_) in the ^1^H NMR spectrum (DMSO-d_6_) (Figure S2, Supplementary Materials). No destruction of the main chain was detected after deprotection.

**Figure 2:**
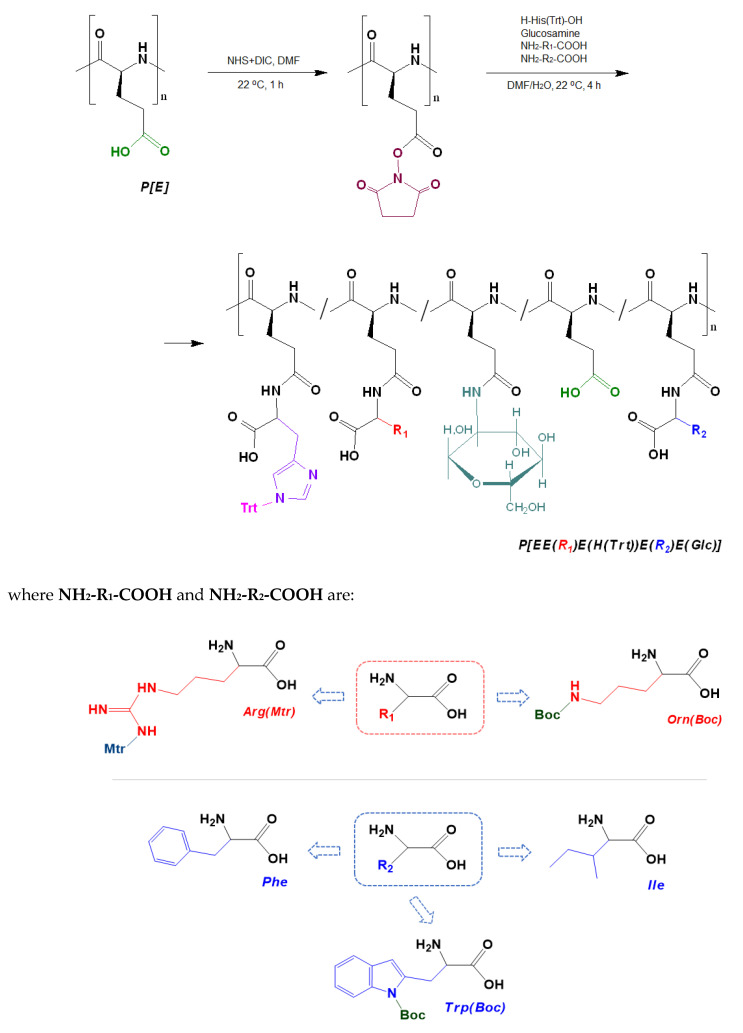
Scheme of the synthesis of amphiphilic copolymers by the post-polymerization modification of poly(α-glutamic acid) and structures of amino acid derivatives used for modification: R_1_ (red moiety) indicates a basic amino acid, R_2_ (blue moiety) indicates a hydrophobic amino acid.

**Figure 3:**
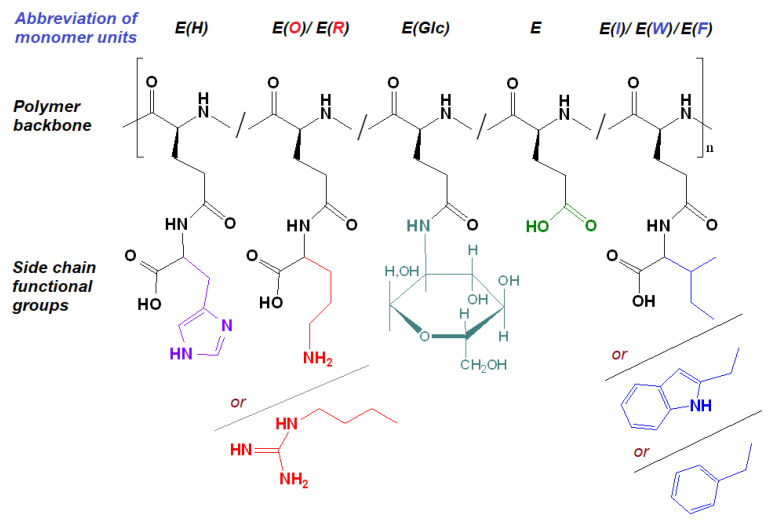
Amphiphilic copolymers obtained after the removal of side protective groups.

The post-modification of P[E] was performed using the method of activated esters. For this, all γ-carboxylic groups of P[E] were activated with the use of *N*,*N*′-diisopropylcarbodiimide (DIC) and *N*-hydroxysuccinimide (NHS) to produce activated *N*-hydroxysuccinimide ester functionality in P[E] (Figure 2). After activation, a four-component mixture of amino-bearing compounds consisting of three amino acids and D-glucosamine was added to the copolymer solution for the reaction. Ornithine (Orn, O), arginine (Arg, R), histidine (His, H) and tryptophan (Trp, W) were used in the protected forms (Figure 2). The final composition of the copolymers and the abbreviation of monomeric units are shown in Figure 3. For the reaction, amino acid derivatives were taken in the following ratio: basic amino acid/histidine/hydrophobic amino acid = 6/3.5/1.5. The mixture of amino acids for the modification was taken in excess, namely, 110 mol% to the number of carboxyl groups. D-Glucosamine (Glc) was taken at 60 mol% of carboxy groups. Since the obtained copolymers did not dissolve in either organic or aqueous media, forming nanoparticles even at low copolymer concentrations, the correct analysis by SEC or static light scattering were not possible. Despite the limited solubility in DMSO-d6, the structures of the obtained polypeptides were analyzed by ^1^H NMR spectroscopy. Signals corresponding to the individual groups within the modifying agents were present in the spectra, but a correct calculation of the composition could not be performed (Figure S3 of Supplementary Materials).

In order to determine the composition of the (glyco)polypeptides obtained, the samples of copolymers were hydrolyzed to free amino acids and glucosamine via the acidic hydrolysis of amide bonds at an elevated temperature (110 °C). This approach is widely used for the amino acid analysis of (poly)peptides [49,50]. The quantitative determination of the free amino acids and glucosamine in a sample was carried out by HPLC analysis (see Section 3.3). The determined composition of the copolymers obtained is presented in Table 1. Despite the 1.7 molar excess of amino-bearing compounds with respect to carboxylic groups, their total modification was in the range of 69–80 mol%. Taking into account that the polymer analogous reactions do not run completely, the obtained results on the modification can be considered successful.

The ratio of the amount of the attached substituent to its initial amount taken for the reaction allows for estimating the fraction of the attached component. In particular, this proportion was 45–53% for the basic amino acids (R or O), 45–57% for histidine, 33–60% for hydrophobic amino acids and 33–38% for glucosamine.

For comparison, Mildner and Menzel reported the modification of poly(α,L-glutamic acid) with D-glucosamine [20]. They found that the degree of modification reached 90 mol% at a loading ratio of 20 mol% Glc per carboxylate but decreased at higher Glc loads. At the same time, the degree of modification was in the range of 39–80 mol% at the loading ratios of 40–100 mol% Glc per carboxylic group.

In our case, the initial amount of glucosamine taken for the reaction was the same as that for arginine and ornithine derivatives, but its coupling efficiency was 1.4 times lower than that for basic amino acids. This may be explained by the worse solubility of glucosamine in the DMF/H_2_O mixture compared to the highly reactive amino acids taken in large amounts. The efficiency of coupling for different amino acids was similar, and the modification by this or that amino acid can be considered random.

In addition, to compare the properties of nanoparticles formed by amphiphilic copolymers obtained by different ways, we modified P[E] with Orn, Phe and the GKH_6_ peptide (H_6_-pept.) as a source of histidine units. The degree of modification was 95% for Orn, 60% for Phe and 75% for modification with the peptide.

With the aim of evaluating the composition of some (glyco)polypeptides by ^1^H NMR spectroscopy, we carried out the sequential modification of P[E] with (1) hydrophobic amino acid, (2) His and Glc and (3) Arg or Orn (Figure S4 of Supplementary Materials). In this case, the number of reagents at each stage corresponded to the amounts used in the complex modification (the number of activated carboxyl groups at each step corresponded to the amount of the used modifier mol%/mol%). It was found that the results obtained by ^1^H NMR spectroscopy (Table S1 of Supplementary Materials) were close to those determined by the HPLC analysis of amino acids.

### 2.2. Preparation and Characterization of Nanoparticles

Depending on the hydrophobic amino acid in the amphiphilic polypeptide, the nanoparticles were prepared due to either self-assembly or nanoprecipitation. In particular, nanoparticles based on Phe- and Ile-containing (glyco)polypeptides were prepared using a previously developed approach based on self-assembly during the slow gradient phase inversion (dialysis) from DMF to water, followed by freeze-drying and final redispersion in the aqueous medium of interest. The use of this approach for Trp-containing polypeptides led to the formation of large aggregates (~500–600 nm). Therefore, in the case of Trp-containing polypeptides, nanoparticles were obtained by nanoprecipitation.

The physicochemical characteristics of nanoparticles, namely, the hydrodynamic diameter, PDI and surface electrokinetic potential (ζ-potential), were measured with the use of dynamic light scattering (DLS), nanoparticle tracking analysis (NTA) and electrophoretic light scattering (ELS). The results obtained are summarized in Table 2.

**Figure 4:**
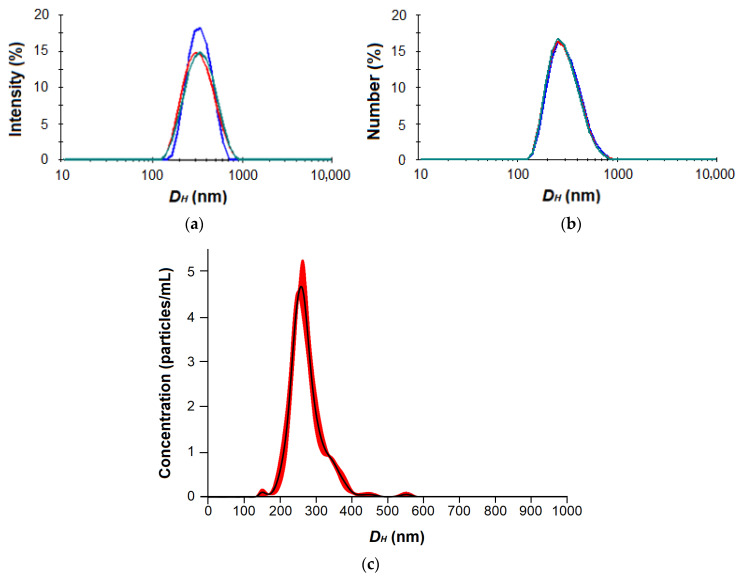
DLS by intensity (**a**) and number (**b**) as well as NTA (**c**) distribution curves for nanoparticles based on P[EE(**R**)E(H)E(**I**)E(Glc)] amphiphilic glycopolypeptide (the color curves reflect multiple measurements for the same sample, *n* = 3).

As can be seen, the average nanoparticle hydrodynamic diameter determined by DLS was larger than the same parameter measured by NTA. This is probably due to the higher sensitivity of NTA to the smaller nanoparticles and the different size averaging. As an example, the typical DLS and NTA distribution curves are shown in Figure 4 and Figure S5 (Supplementary Materials).

In general, all of the prepared nanoparticles had a hydrodynamic diameter in the range of 200–350 nm according to NTA and of 230–400 nm according to DLS. In the case of Phe-containing nanoparticles, the hydrodynamic diameter was decreased from 332 to 229 nm (DLS) when the amount of Phe was increased from 5 to 9 mol%. A similar result was observed earlier for random copolymers of glutamic acid and phenylalanine (P[Glu-co-Phe]) obtained by the ROP of NCAs [29]. In particular, increasing the amount of Phe from 15 to 21 mol% contributed to the decrease in *D_H_* from 320 to 230 nm (pH 7.4). Such compaction of nanoparticles is a consequence of the hydrophobic interactions of phenylalanine residues in aqueous media. Due to their poor solubility, Phe residues tend to hide inside the particles and contribute to their stabilization by the π–π-interactions between aromatic moieties. Despite the larger amount of hydrophobic amino acid in the previously developed P[Glu-co-Phe]-based nanoparticles, the Phe-containing glycopolypeptide nanoparticles developed in this work had comparable sizes. This can be explained by the contribution of electrostatic interactions additionally occurring in nanoparticles self-assembled from polypeptides containing both negatively and positively charged amino acids. It is known that polyelectrolyte interactions contribute to macromolecule compaction, and this property is successfully used in the delivery of nucleic acids with cationic polymers [52]. Recently, this effect was also demonstrated for nanoparticles self-assembled from amphiphilic random polypeptides containing Lys, Glu and Ile and prepared by the ROP of NCAs. In particular, increasing the amount of Glu from 16 to 31 mol% while reducing the amount of Phe from 18 to 12 mol% contributed to more compact nanoparticles (size reduction of 50 nm) due to the combined effect of hydrophobic and electrostatic interactions [30].

Ile-containing (glyco)polypeptide-based nanoparticles with similar compositions also exhibited similar hydrodynamic diameters. At the same time, despite the similar composition for Trp-based polypeptides, their nanoparticles differed quite significantly in *D_H_*. However, it should be taken into account that Ile-containing nanoparticles were self-assembled, while the Trp-containing ones were obtained by nanoprecipitation. Despite the large number of hydrophobic and positively charged amino acids, the largest hydrodynamic diameter was observed for H_6_-peptide-containing polymers. This may be due to the exposure of the grafted hydrophilic H_6_-peptide moieties from the surface to water that contributes to *D_H_*.

Among the nanoparticles obtained, the lowest PDI was detected for nanoparticles prepared from Ile-conaining polypeptides. Compared to Phe and Trp, Ile is less hydrophobic, and its polypeptides have better solubility, leading to a narrower dispersion. All nanoparticles had a negative surface charge with an absolute ζ-potential of about 40–46 mV (Table 2 and Figure S6, Supplementary Materials).

### 2.3. Paclitaxel Loading and Characterization of Nanoformulations

It is known that PTX demonstrates a high efficiency in the treatment of breast, lung, ovary and bladder cancers [44]. The use of high doses of paclitaxel (PTX) in medical practice is associated with the low bioavailability of this drug due to its high hydrophobicity and poor solubility in biological fluids. The use of conjugates and nanoformulations of paclitaxel improves its bioavailability and minimizes side effects. This is confirmed by the fact that several (bio)polymeric PTX formulations are commercially available today [53].

The PTX nanoformulations were prepared using the previously developed approach [38]. Briefly, a mixture of PTX and a copolymer dissolved in DMSO was lyophilized to achieve high encapsulation efficacy. Then, the freeze-dried nanoformulations were dispersed in the medium of interest (deionized water or buffer) under short-term ultrasonication. The use of an initial amount of 50 µg of PTX per mg of polymer provided a total loading of the drug (a 5 wt% loading). An increase in the initial PTX amount to 100 and 200 µg of PTX/mg of polymer was accompanied by 98–99% and 80–92% loading efficacy, respectively (the 9.8–9.9 and 16.0–18.4 wt% loading, respectively). According to the published data, the loading of PTX of 1.5–15 wt% is considered appropriate for the in vivo performance of the nanoformulations [54,55,56]. For further physicochemical and biological evaluation, formulations containing 50 μg of PTX per mg of polymer were selected due to both the high encapsulation efficacy and sufficient drug loading.

As empty nanoparticles, the PTX nanoformulations were also characterized to determine their hydrodynamic diameter, PDI and surface ζ-potential. The obtained results are presented in Table 3. It can be seen that PTX loading contributed to a decrease in the hydrodynamic diameter, with a slight increase in PDI. According to DLS, the hydrodynamic diameters of nanoformulations based on glycopolypeptides were in the range of 170–290 nm. In general, a reduction in *D_H_* by 30–110 nm was observed. The most considerable reduction in *D_H_* was observed for delivery systems based on Trp-containing glycopolypeptides. The reason for this may be the differences in the methods for obtaining empty Trp-containing nanoparticles (nanoprecipitation) and other nanoparticles (self-assembly) and the same method for preparing loaded nanoparticles. As an example, the distribution curve (NTA) for PTX-loaded nanoparticles based on P[EE(R)E(H)E(I)E(Glc)] is shown in Figure S7 (Supplementary Materials).

In general, the decrease in the hydrodynamic diameter of PTX-loaded nanoparticles may be the result of nanoparticle compaction due to hydrophobic interactions between the highly hydrophobic PTX and hydrophobic amino acids. Moreover, PTX containing three hydroxyl groups per molecule can additionally form hydrogen bonds with the carboxylated copolymer. Thus, hydrophobic interactions and hydrogen bonds between the drug and polymer contribute to the formation of a quite stable nanoformulation.

**Figure 5:**
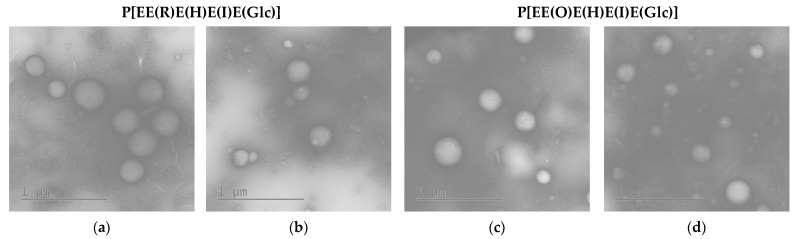
TEM images of empty (**a**,**c**) and PTX-loaded (**b**,**d**) nanoparticles based on P[EE(**R**)E(H)E(**I**)E(Glc)] (**a**,**b**) and P[EE(**O**)E(H)E(**I**)E(Glc)] (**c**,**d**).

The morphology of the empty and PTX-loaded nanoparticles was assessed by transmission electron microscopy (TEM) with uranyl acetate staining (Figure 5). As expected, the obtained nanoparticles were spherical. For comparison, the average diameter ($\bar{D}$) of empty nanoparticles in a dry state was calculated from TEM images. This parameter was found to be 300 ± 52 nm (PDI = 0.09) and 262 ± 70 nm (PDI = 0.21) for nanoparticles based on P[EE(R)E(H)E(I)E(Glc)] and P[EE(O)E(H)E(I)E(Glc)], respectively. The obtained values were 10–20% lower than the hydrodynamic diameters obtained by DLS for the same nanoparticles in dispersion (see Table 2). This means that the obtained nanoparticles have quite dense packing and did not considerably shrink after drying. A similar effect was recently established for nanoparticles produced from amphiphilic polypeptides synthesized by ROP and containing oppositely charged amino acids [28,30]. In contrast, nanoparticles self-assembled from amphiphilic polypeptides which contain only positively or negatively charged amino acids were quite soft and demonstrated a reduction in size by 2–3 times after drying [29].

As in the case of DLS results, the average diameter of the PTX-loaded nanoparticles was also lower. According to TEM, the $\bar{D}$ values for PTX nanoformulations were 210 ± 56 (PDI = 0.22) for the P[EE(R)E(H)E(I)E(Glc)] delivery system and 185 ± 59 (PDI = 0.31) for the P[EE(O)E(H)E(I)E(Glc)] delivery system. In this case, the particle size values were 20–25% lower than the *D_H_* values obtained by DLS. Such a reduction can be associated with the water depletion upon the sample drying on a grid.

Summarizing the results of DLS and TEM obtained for prepared PTX nanoformulations, it can be concluded that the developed systems meet the size requirements (≤300 nm) for delivery systems considered for parenteral administration [57,58].

**Figure 6:**
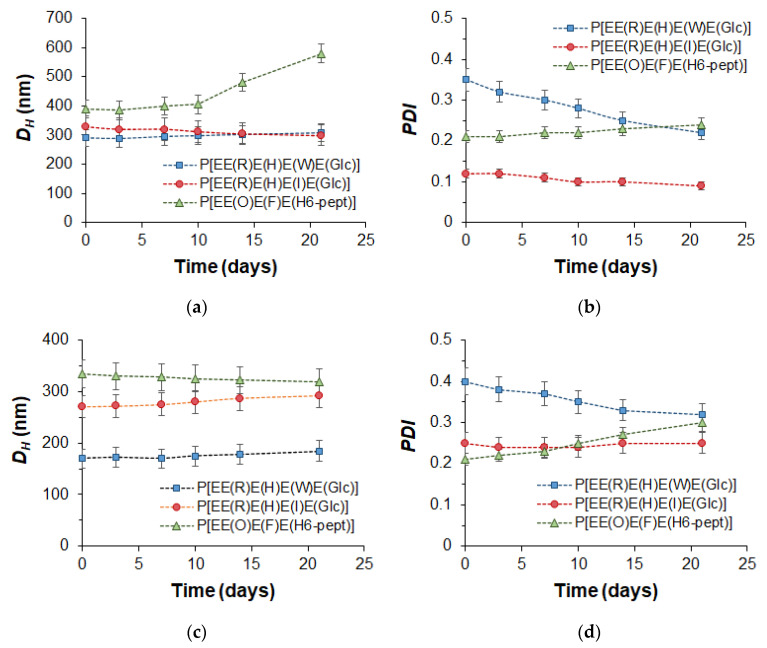
Monitoring of the storage stability of the empty (**a**,**b**) and PTX-loaded (**c**,**d**) nanoparticles (DLS; 20 °C, H_2_O): hydrodynamic diameter (*D_H_*) (**a**,**c**) and PDI (**b**,**d**).

The stability of empty nanoparticles and their PTX formulation was examined using DLS to monitor changes in the hydrodynamic diameter and PDI during the incubation of nanoparticles in water at room temperature (20 °C) for 3 weeks. The results presented in Figure 6 clearly indicate that, with the exception of the empty P[EE(**O**)E(**F**)E(**H_6_-pept**)] nanoparticles, the other nanoparticles and their PTX compositions retained their hydrodynamic diameter during the experiment. As for PDI, for most systems, this parameter was retained, but for Trp-containing polypeptides, it decreased during the monitoring time. Given that Trp-containing nanoparticles were prepared by non-equilibrium nanoprecipitation, the decrease in PDI over time for this kind of nanoparticle may be related to the intraparticle self-reorganization of polymer chains for achieving more favorable conformation.

### 2.4. Biological Evaluation of Nanoparticles

PTX is used in the therapy of several types of cancer, e.g., breast, ovarian, lung and Kaposi sarcoma, and in combined therapy for some other solid tumors [59]. In this study, to evaluate the cytostatic effect of PTX nanoformulations, we selected lung cancer cells.

**Figure 7:**
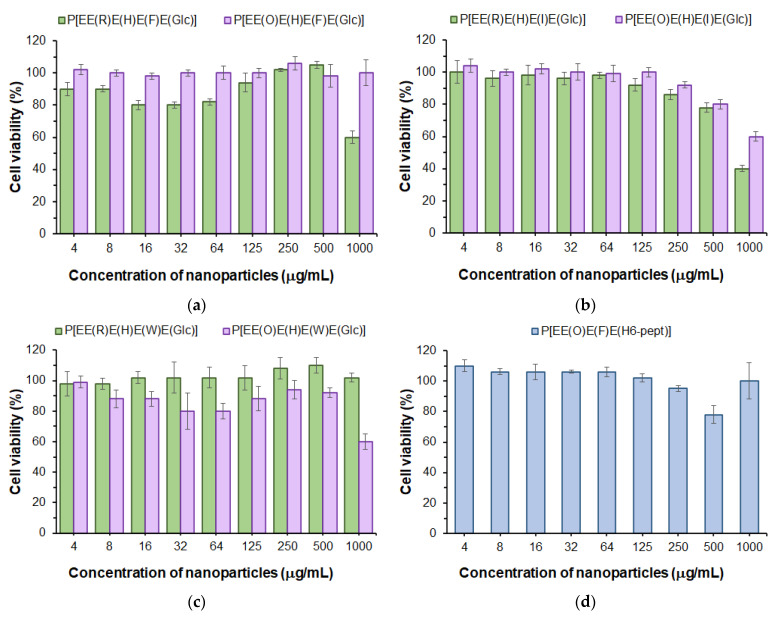
Viability of BEAS-2B cells in the presence of nanoparticles based on Phe- (**a**), Ile- (**b**) and Trp-containing glycopolypeptides (**c**) as well as nanoparticles self-assembled from a polypeptide modified with H_6_-peptide (**d**) (CTB assay, 72 h).

First, in order to evaluate the cytotoxicity of the developed polypeptide nanoparticles to normal cells, we used the human lung epithelial cell line (BEAS-2B). The examination of cell viability in the presence of nanoparticles was performed with the use of the CellTiter-Blue (CTB) assay in a range of concentrations from 4 to 1000 μg/mL for 72 h. As can be seen in Figure 7, a viability of BEAS-2B cells above 75% was observed for all kinds of (glyco)polypeptide nanoparticles at concentrations up to 500 μg/mL and, for some of them, even up to 1000 μg/mL. Comparing Phe- and Ile-containing polypeptides, one can detect a slightly greater cytotoxicity of Arg-containing nanoparticles relative to the same Orn-containing analogs (Figure 7a,b). In turn, for Trp-containing polypeptide nanoparticles obtained by nanoprecipitation, there was another tendency (Figure 7c). This may indirectly indicate a similar arrangement of (glyco)polypeptides in nanoparticles during self-assembly and a different one for nanoprecipitated polymers. Nanoparticles self-assembled from P[E] modified with Orn, Phe and the H_6_-peptide were nontoxic over the entire concentration range tested (Figure 7d). Despite the high proportion of positively charged amino acids in polypeptides (27–38 mol%), the low cytotoxicity of all nanoparticles is in agreement with their physicochemical characteristics, particularly the negative surface charge. This may indicate that these amino acids are inside the nanoparticles and are involved in intraparticle stabilization but practically do not affect the surface properties.

The obtained results are also in agreement with our previous findings. Negatively charged Glu-based random polypeptides obtained by the ROP of NCAs demonstrated a low cytotoxicity to normal cells [29,35] (IC_50_ > 500 μg/mL). It is also known that the modification of cationic polypeptides with His-units reduces their cytotoxicity [60].

One of the important properties of delivery systems is their reduced uptake by macrophages, which contributes to lowering their rapid elimination from the blood due to phagocytosis. It is known that negatively charged delivery systems are less prone to elimination from the blood than positively charged ones [61]. In this study, flow cytometry was applied to evaluate the uptake of the developed nanoparticles by macrophages. Before the analysis, mouse macrophages (J774A.1 cell line) were incubated with Cy5-labeled nanoparticles (5 μg dye/mg polymer) for 5 h. Cy5-labeled nanoparticles based on poly(D,L-lactide) (PLA) (*D_H_* = 140 nm, *PDI* = 0.09, ζ-potential = −37.5 mV) obtained by nanoprecipitation were used as a benchmark. The selection of PLA as the control polymer nanomaterial was motivated by the wide application of PLA nano- and microparticles as delivery systems for various drugs, including their clinical use [62]. In addition, self-assembled nanoparticles obtained from the amphiphilic copolymer of N-vinyl succinamic acid and O-cholesteryl acrylate (P[VSAA-*co*-ChA]) (*D_H_* = 250 nm, *PDI* = 0.3, ζ-potential = −27 mV) were included in the study for comparison. This copolymer had a random structure and provided self-assembled nanoparticles with a negative surface charge and a hydrodynamic diameter close to that of the polypeptide nanoparticles developed in this work. The accumulation of nanoparticles was calculated as a percentage of the relative fluorescence intensity value obtained for Cy5-PLA nanoparticles.

**Figure 8:**
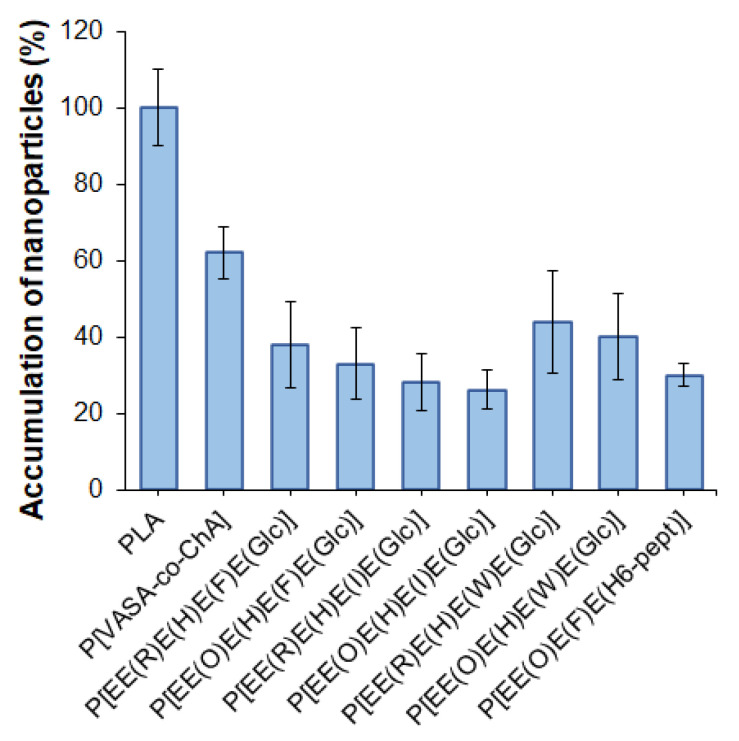
Accumulation of nanoparticles by macrophages after 5 h of co-incubation.

It is known that the uptake rate of nanoparticles depends on their surface properties. Positively charged nanoparticles are captured faster than negatively charged or neutral ones [63]. All of the studied nanoparticles (including the control ones) were negative, so in our case, the surface charge cannot be a driving force for the accelerated uptake. The results of the accumulation of various nanoparticles by macrophages are shown in Figure 8.

The most intense uptake by macrophages was observed for PLA-based nanoparticles, whereas the accumulation of nanoparticles based on amphiphilic P[VASA-co-ChA] and (glyco)polypeptides was less pronounced (*p* < 0.005). The accelerated accumulation of PLA nanoparticles inside macrophages can be explained by their better penetration ability due to their smaller size compared to other nanoparticles [63]. Indeed, those almost twice the size of P[VASA-co-ChA] nanoparticles showed only a 60% cellular uptake compared to the PLA ones (*p* < 0.005). At the same time, the (glyco)polypeptide nanoparticles showed a 28–45% accumulation relative to PLA nanoparticles.

### 2.5. Cytostatic Effect of Paclitaxel Nanoformulations

The human lung adenocarcinoma cell line (A549) was selected to examine the cytostatic effect of PTX nanoformulations. Free PTX (stock solution in DMSO) and its commercial formulation, namely, Paclitaxel LANS (PTX-LANS), representing the ethanol solution also containing macrogol glyceryl hydroxystearate and citric acid, were used as controls.

According to the literature, the IC_50_ for free PTX is usually between 1.1 and 8.5 ng/mL (1.3–10 nM) [64,65,66]. In our case, the IC_50_ values determined for PTX and PTX-LANS were 0.8 and 2.0 ng/mL, which, considering the SD values, are in good agreement with the literature data. The IC_50_ values determined for the developed nanoformulations and control drug solutions are provided in Table 4 and Figure S8 (Supplementary Materials).

In general, the IC_50_ values for PTX nanoformulations indicate their high cytotoxic activity towards cancer cells (1.3–7.8 ng/mL). The higher IC_50_ values determined for PTX nanoformulations can be explained by the gradual accumulation of PTX in the medium due to the drug release as opposed to free PTX, whose concentration in the medium is initially defined and constant. The results obtained in this study are similar to those of the previously reported PTX delivery systems based on glycopolymers [31] or PEGylated phospholipid microparticles [67].

Thus, PTX delivery systems based on the synthesized biodegradable (glyco)polypeptides had a high activity against cancer cells, while the empty polypeptide nanoparticles were not cytotoxic to the normal cells. Moreover, such nanoparticles demonstrated the reduced uptake by macrophages. At the same time, the use of delivery systems can help to overcome the problem of the poor solubility of PTX and increase its bioavailability.

## 3. Materials and Methods

### 3.1. Chemicals, Supplements and Biologicals

L-glutamic acid γ-benzyl ester (≥97%), L-phenylalanine (≥98%), L-isoleucine (≥98%), 1-tert-butoxycarbonyl-L-tryptophan (≥98%), triphosgene (98%), α-pinene (98%), trifluoroacetic acid (TFA, 99%), trifluoromethanesulfonic acid (TFMSA, 99%), *N*,*N*′-Diisopropylcarbodiimide (DIC, 99%), *N*-hydroxysuccinimide (NHS, 98%) and dimethyl sulfoxide-d6 (DMSO-d6, 99.8%) were purchased from Sigma–Aldrich (Darmstadt, Germany) and used as received. N′-trityl-*L*-histidine (98%), δ-tert-butoxycarbonyl-L-ornithine (98%) and N′-(4-methoxy-2,3,6-trimethylphenyl-sulfonyl)-L-arginine were the products of Alfa Aesar GmbH (Kandel, Germany), Bachem AG (Bubendorf, Switzerland) and Iris Biotech (Marktredwitz, Germany). D-Glucosamine (98%) was a product of BLDPharm (Shanghai, China).

*N*,*N*-dimethylformamide (DMF), 1,4-dioxane, dimethyl sulfoxide (DMSO), diethyl ether, ethyl acetate, petroleum ether and other organic solvents were purchased from Vecton Ltd. (St. Petersburg, Russia), and purified and dried before use according to standard protocols. The salts used for the preparation of buffer solutions were of analytical purity grade. The formic acid (98%, ACS) and acetonitrile (Ultra Gradient HPLC Grade) used for HPLC analysis were received from PanReac (Barcelona, Spain) and J.T.Baker (Philipsburg, NY, USA), respectively. The GKH_6_ peptide was synthesized in IMC RAS by solid-state peptide synthesis using the Fmoc-strategy and Rink Amide AM resin using the standard protocol.

All buffers were filtered using membrane filters (0.22 μm) produced by Millipore Sigma (St. Luis, MO, USA). The purification of the synthesized polymers was carried out with the use of Spectra/Pore^®^ dialysis bags (MWCO:1000, Rancho Dominguez, CA, USA) and Orange Scientific dialysis bags (MWCO: 2000) (Braine-l’Alleud, Belgium).

Human lung carcinoma cells (A549), human lung epithelial cells (BEAS-2B) and mouse macrophages (J774A.1) were purchased from CLS Cell Lines Service GmbH (Eppelheim, Germany). Dulbecco’s Modified Eagle’s Medium (DMEM, Merck, Darmstadt, Germany) was used for the cultivation of A549 and J774A.1 cells, while LHC-9 (Thermo Fisher Scientific, Dreieich, Germany) medium was used for the cultivation of BEAS-2B cells. Both media were supplemented with 10% (*v/v*) fetal calf serum (FCS) (Biochrom, Berlin, Germany) and 1% (*v/v*) penicillin/streptomycin (P/S) (Biochrom, Berlin, Germany). The CellTiter-Blue assay reagent was a product of Promega (Madison, WI, USA). Cyanine-5 amine (Cy_5_-NH_2_, 95%) was received from Lumiprobe (Moscow, Russia).

All other materials are described further upon their appearance in the text.

### 3.2. Polymerization and Polymer Post-Modification

E(OBzl) NCA was synthesized as described earlier [29]. Freshly synthesized, purified and dried monomer was polymerized by ROP, using *n*-hexylamine as an initiator. For polymerization, 4 wt% monomer solution in freshly distilled and anhydrous 1,4-dioxane was incubated at 30 °C for 72 h. The monomer/initiator ratio was 100. The polymerization was carried out in an argon atmosphere. The reaction product was precipitated and washed by diethyl ether and then air-dried. The polymer yield was 75%.

To remove the benzyl protection of the γ-carboxyl group of glutamic acid, 200 mg of the polymer was dissolved in 4 mL of trifluoroacetic acid in an ice bath under stirring. Then, 200 μL of trifluoromethanesulfonic acid was added to the resulting solution, allowed to react under cooling for 20 min and then incubated at room temperature for 4 h. To complete the reaction, the resulting polymer was precipitated in a fivefold excess of cooled diethyl ether, the precipitate was separated from the supernatant by centrifugation and then dissolved in 10 mL of DMF and the resulting solution was transferred to a dialysis bag with a molecular weight cutoff (MWCO) of 1000. Dialysis was performed sequentially against DMF/deionized water mixture (50/50, *v/v*), deionized water, 1 M sodium chloride solution and deionized water. Then, the obtained polymer was freeze-dried. The removal of the protective groups was monitored by the ^1^H NMR spectroscopy (Figure S2 of Supplementary Materials).

The modification of P[E] was performed by the preliminary activation of 100% carboxyl groups in the polymer using DIC and NHS. P[E] was dissolved in DMF, a solution of NHS in DMF (twofold molar excess over the amount of carboxyl groups) was added to the polymer solution under stirring. After 15 min, a solution of DIC in DMF (1.1-fold molar excess over the amount of carboxyl groups) was added to P[E]/NHS-containing reaction mixture. The concentration of the polymer in the final reaction solution was 15 wt%. The reaction was left under stirring for 1 h at 22 °C.

Amino acid derivatives were taken in the following ratio: basic amino acid/histidine/hydrophobic amino acid = 6/3.5/1.5. The mixture of amino acids for the modification was taken in excess, namely, 110 mol% to the amount of carboxyl groups. Glucosamine was taken at 60 mol% of carboxy groups. The derivatives of ornithine, tryptophan, histidine and arginine as well as isoleucine were dissolved in DMF. In turn, glucosamine and phenylalanine were dissolved in water. To prepare the target reaction mixtures, the calculated aliquots of amino acid derivatives, glucosamine and, if necessary, phenylalanine were added to the solutions of activated P[E] and mixed. Thus, the modification was carried out in DMF/water solutions with following ratio of solvents (*v/v*): DMF/water = 70/30 for systems containing phenylalanine and 80/20 for systems containing tryptophan and isoleucine as hydrophobic acid. After 4 h of stirring at 22 °C, the reaction mixture was transferred to a dialysis bag (MWCO 2000). Dialysis was performed sequentially against DMF, DMF/deionized water mixture (50/50, *v/v*), deionized water, 1 M sodium chloride solution and deionized water again for two days. The resulting polymers were freeze-dried. The yields of the modified polymers were in the range of 77–89%.

To remove the Mtr-protecting group of Arg, the polypeptides were dissolved in TFA, and then TFMSA was added to the solution as described above for the deprotection of P[E]. Polypeptides containing Arg(Mtr) were deblocked for 3 h. The Trt- and Boc-protective group was removed by TFA for 1.5 h. Trp-containing polymers were deprotected in an argon atmosphere. After deprotection, the polymers were purified by dialysis using the scheme given above.

### 3.3. Characterization of Polymers

The parent polymer (P[E(OBzl)]) was analyzed by SEC using the Shimadzu LC-20 Prominence system equipped with a refractometric RID 10-A detector (Kyoto, Japan) and a Styragel Column, HMW6E (7.8 mm × 300 mm, 15–20 µm bead size, Waters, Milford, MS, USA). The analysis was performed using 0.1 M LiBr in DMF as an eluent at a flow rate of 0.3 mL/min at 40 °C. Calculations of *M_w_*, *M_n_* and *Ɖ* were made with the use of the GPC LC Solutions software (Shimadzu, Kyoto, Japan) relative to a preliminary built calibration curve for poly(methyl methacrylate) standards with M*_w_* 17,000–250,000 (*Ð* ≤ 1.14).

^1^H NMR spectroscopy of the synthesized polymers was performed using a Bruker AC-400 NMR spectrometer (400 MHz) (Karlsruhe, Germany) at 25 °C in DMSO-d_6_. Some spectra and the description of signals can be found in Figures S3 and S4 (Supplementary Materials).

The composition of polypeptides was estimated using the quantitative HPLC analysis of free amino acids obtained after the total acidic hydrolysis of copolymers. For this, 2 mg of the copolymer was placed into the ampoule, and a 6 M HCl solution containing 0.0001% phenol was added in the ratio of 1 mL/0.5 mg of the copolymer. Then, the ampoule was filled with argon and sealed, and the mixture was incubated at a temperature of 110 °C for 4 d. After hydrolysis, the solution was evaporated several times with deionized water until a neutral pH was attained. Finally, the evaporated residue was diluted with 0.5 mL of water, quantitatively transferred to a glass and freeze-dried. The quantitative HPLC analysis of amino acids was performed at the Chemical Analysis and Materials Research Center of Research Park of St. Petersburg State University using a known method based on the pre-column derivatization of amino acids with 5-dimethylaminonaphthalene-l-sulfonyl chloride (dansyl chloride) [68,69]. The analysis was performed using the Shimadzu LC-30 Nexera ultrafast liquid HPLC system equipped with an SPD-M20A diode-matrix detector (Tokyo, Japan) and a Kinetex^®^ Phenomenex C18 column (150 mm × 2.1 mm, 5 µm bead size). Deionized water prepared by a D-301 deionizer (Aquilon, Russia) and containing 0.1% formic acid (phase A) and acetonitrile (phase B) were used as eluents. The analysis was carried out at a flow rate of 0.5 mL/min and at 40 °C using the following gradient: 0–1 min—10% B, 1–16 min—from 10 to 95% B, 16–18 min—95% B. The detection was performed at 254 nm. The data were processed using Shimadzu LabSolutions Software, version 5.96 (Tokyo, Japan). The quantities of certain amino acids were calculated using preliminary built calibration curves. Some chromatograms and calibration curves are shown in Figure S9 (Supplementary Materials).

### 3.4. Preparation and Characterization of Nanoparticles

Except for the Trp-containing polymers, the preparation of nanoparticles included several steps: (1) self-assembly during the slow gradient phase inversion (dialysis) from DMF to water; (2) freeze drying and (3) redispersion in the aqueous media of interest. Dried nanoparticles can be stored at 4 °C and redispersed prior to use. Usually, nanoparticles are redispersed in 0.01 M sodium phosphate or sodium borate buffer (pH 7.4) at a concentration of 2 mg/mL. Trp-containing polymers were prepared by nanoprecipitation. For this, polymers were preliminary dissolved in DMSO (2 mg per 100 μL DMSO), and then the obtained solution was dropped to deionized water/PBS (900 μL) under intensive stirring (1000 rpm). The obtained dispersion was additionally ultrasonicated using a Sonopuls ultrasound probe (30%, 30 s) and left in the cold for 30 min. For characterization, stock solutions were diluted with deionized water to 0.2 mg/mL for DLS/NTA or 0.5 mg/mL for TEM analysis.

DLS and ELS were used to measure the hydrodynamic diameter (*D_H_*), the polydispersity index (PDI) and the ζ-potential of nanoparticles by a ZetasizerNano-ZS (Malvern, UK) equipped with a He–Ne laser at 633 nm at a scattering angle of 173° and a temperature of 25 °C. The measurements of *D_H_* by DLS were performed in a range of sizes from 10 nm to 10 µm. In addition, the *D_H_* of nanoparticles was analyzed by NTA using NanoSight NS300 (Malvern, UK). The measurements of *D_H_* by NTA were performed in a range of sizes from 10 nm to 1 µm. For measurements, the colloid solutions with a concentration of 0.1–0.2 mg/mL in water or PBS were used.

The morphology of nanoparticles as well as their average diameter in a dry state were determined by TEM (Jeol JEM-2100, Kyoto, Japan). The analysis was carried out using the 300 mech Cu-grids covered with carbon and formvar. Before the analysis, a colloid solution (0.5 mg/mL, in water) was dropped at the surface of a grid and dried. Then, the grid was treated with 2% (*w/v*) uranyl acetate solution for 30–60 s. The excess of the contrasting agent was quickly blotted, and the grid was left for 24 h at room temperature and then analyzed. The average diameter of the particle was calculated using ImageJ open software developed by the National Institute of Mental Health (Bethesda, MD, USA).

### 3.5. Drug Loading

PTX was loaded as described elsewhere [38]. Briefly, a 50–200 μL solution of PTX in DMSO (1.0 mg/mL) was added to a 400 μL solution polymer in DMSO (1.0 mg/mL). The resulting mixture was ultrasonicated for 20 s and freeze dried. After that, the lyophilizate of the resulting formulation was dispersed in 0.01 M PBS, pH 7.4, under ultrasonication for 60s. The characteristics of all the formed formulations were examined by the DLS and ELS. To determine the amount of free paclitaxel, the dispersion was purified with the use of the ultrafiltration tubes with a molecular weight cutoff membrane of 5000 using a mixture of acetonitrile:water (1:10). The collected filtrates were freeze-dried and dissolved in 100 μL of acetonitrile. The quantification of paclitaxel was performed by reversed-phase HPLC with UV detection (λ = 237 nm) using Shimadzu Prominence LC-20AD chromatographic systems (Tokyo, Japan) equipped with a diode-matrix detector and a Grace Smart C18 column (particle size 5 μm, column size 4.6 mm × 150 mm). The flow rate of the mobile phase was 0.5 mL/min, and the sample introduction loop volume was 20 µL. Water and acetonitrile were used as eluents A and B. The analysis of elution was performed by the gradient elution: 0–15 min—0–100% eluent B.

### 3.6. Biological Evaluation

A549 cancer cells were cultivated in Dulbecco’s Modified Eagle’s medium, while BEAS-2B normal cells were grown in LHC-9 medium, both containing 10% (*v/v*) fetal calf serum (FCS) and 1% penicillin/streptomycin. All types of cells were cultivated in a humidified environment at 37 °C and 5% CO_2_, changing the medium three times per week.

#### 3.6.1. Cell Viability Assay

In total, 4 × 10^3^ cells per well were seeded in a 96-well plate (100 μL/well) in an appropriate culture medium and cultivated under a humidified atmosphere of 5% CO_2_ at 37 °C. After 24 h, the medium was replaced with the culture medium containing test nanoparticles of different concentrations (from 4 to 1000 µg/mL). The cell viability was determined after 72 h of incubation using the CTB assay [38]. The data obtained were expressed as a percentage of the control (cells incubated without particles).

#### 3.6.2. Capture of Nanoparticles by Macrophages

Before testing, nanoparticles were labeled with the use of Cy5-NH_2_ dye. At the first step, a 20 μL NHS solution in DMSO with a concentration of 0.3 mg/mL (6 μg, 52 nmol) was added to a 960 μL dispersion of nanoparticles in water containing 1.0 mg of polymer nanoparticles and left for 10 min under stirring. At the second step, a 10 μL EDC solution in DMSO with a concentration of 0.3 mg/mL (3 μg, 19 nmol) was added to the mixture at the first step and left for 50 min under stirring. Finally, a 10 μL Cy5-NH_2_ in DMSO with a concentration of 0.5 mg/mL (5 μg, 8 nmol) was mixed together with the nanoparticles’ dispersion under stirring, and the reaction medium was left for 2 h. To check the labeling efficacy, the dispersions were purified by ultrafiltration from the unbound dye. The quantification of the unbound dye was carried out spectrophotometrically at a wavelength of 650 nm using the preliminary bound calibration curve. The labelling efficacy was 98–100%.

For the evaluation of the particles’ cellular uptake efficacy, flow cytometric analysis was used. In total, 4 × 10^4^ J774A.1 cells per well were seeded in a 24-well plate (500 μL/well) in Dulbecco’s Modified Eagle’s medium and cultivated under a humidified atmosphere of 5% CO_2_ at 37 °C. After 24 h of cultivation, the medium was removed, and 1.5 mL of the medium containing Cy5-labled polymer particles (25 μg/mL) was added. The exposure time was 5 h. Then, the cells were washed with warm PBS, removed into Eppendorf’s, centrifuged at 300 rpm for 5 min and resuspended in 200 µL of PBS. The fluorescence of the samples was analyzed by the flow cytometer BD Accuri C6 with a 488 nm Argon-ion laser. Cy5 fluorescence was collected by a 585/40 band-pass filter. At least 10,000 events per sample were analyzed. Only viable cells were taken for the analysis. The data were normalized with respect to the control, i.e., to the fluorescence intensity of cells incubated without particles. The accumulation value for each sample was calculated as a percentage of the relative fluorescence intensity value obtained for Cy5-PLA-based nanoparticles. 

#### 3.6.3. Study of the Cytostatic Effect of Free Drug and Drug-Loaded Nanoparticles

The experiment was carried out as described in Section 3.6.1 but with free PTX or PTX-loaded nanoparticles as testing materials and A549 cells. The study examined a range of concentrations from 0.625 to 1000 ng/mL relative to PTX. Concentration-dependent normalized cell viability data obtained from the CTB assay were fitted by using non-linear curve fitting/growth/sigmoidal/dose–response fitting functions (OriginPro 8.6). IC_50_ values were calculated from the fitted dose–response curves.

### 3.7. Statistics

All measurements of physicochemical characteristics were performed three times. The calculation of the average particle diameter was performed using three images of the same type of particle for not less than 30 nanoparticles. All biological experiments were performed for four replicates of the same sample. The data were processed with the use of Excel MS Software. All values are given as the mean ± SD. One-way ANOVA was used to distinguish the statistical significance between groups. When *p* < 0.05, the results were considered statistically significant.

## 4. Conclusions

In this work, we demonstrated the possibility of the successful synthesis of amphiphilic (glyco)polypeptides by post-polymerization modification. The obtained copolymers were suitable for the preparation of nanoparticles and hydrophobic drug delivery systems based on them. Due to the negatively charged surface, the obtained nanoparticles were non-toxic to human lung epithelial cells (BEAS-2B) and exhibited reduced macrophage uptake. An examination of the cytostatic activity of the paclitaxel nanoformulations of 200–330 nm demonstrated a high efficacy (IC_50_ = 1.3–7.8 ng/mL) against human lung adenocarcinoma cells (A549). Thus, the developed nanoformulations can allow for overcoming the poor solubility of PTX and at the same time increase its bioavailability. In general, the properties of the obtained nanoparticles and the paclitaxel delivery systems based on them were similar to those obtained by copolymerization methods. At the same time, post-polymerization modification is a simple and non-laborious approach that allows for the ease of the introduction of various functional moieties into the poly(amino acid) main chain.

## Figures and Tables

**Table 1:** Composition of the amphiphilic copolymers, as determined by quantitative HPLC amino acid analysis.

Sample	Modifier					Unmodified *E* Units ** (mol%)
	Determined Composition (mol%) *					
	*R* or *O*	*H*	*Glc*	*F*, *I* or *W*	*H_6_-pept.*	
P[EE(**R**)E(H)E(**F**)E(Glc)]	28	16	20	5	−	31
P[EE(**O**)E(H)E(**F**)E(Glc)]	32	18	21	9	−	20
P[EE(**R**)E(H)E(**I**)E(Glc)]	29	19	19	8	−	25
P[EE(**O**)E(H)E(**I**)E(Glc)]	29	16	21	6	−	28
P[EE(**R**)E(H)E(**W**)E(Glc)]	31	19	22	5	−	23
P[EE(**O**)E(H)E(**W**)E(Glc)]	27	20	23	6	−	24
P[EE(**O**)E(**F**)E(H_6_-pept)]	38	−	−	18	1.5	41.5

* Calculated from the results of the quantitative HPLC analysis for total copolymer hydrolysates. Side-chain amino acids and glucosamine were calculated relative to glutamic acid, taken as 100%. The data presented represent the average values obtained from two independent syntheses and HPLC analyses. The deviation in modifications for individual components was 3–10 mol%. ** Calculated as the difference between the total number of glutamic acid units (100%) and the number of modified units.

**Table 2:** Characteristics of nanoparticles obtained from the synthesized amphiphilic polypeptides.

Sample	DLS			NTA		ζ-Potential (mV)
	*D_H_* (nm) (by Intensity)	*D_H_* (nm) (by Number)	PDI *	*D_H_* (nm)	PDI **	
P[EE(**R**)E(H)E(**F**)E(Glc)]	330	−	0.30	315	0.24	−46.3 ± 1.3
P[EE(**O**)E(H)E(**F**)E(Glc)]	230	−	0.35	195	0.11	−45.5 ± 2.1
P[EE(**R**)E(H)E(**I**)E(Glc)]	330	290	0.12	275	0.10	−42.8 ± 2.5
P[EE(**O**)E(H)E(**I**)E(Glc)]	325	280	0.13	265	0.10	−45.7 ± 0.9
P[EE(**R**)E(H)E(**W**)E(Glc)]	290	230	0.35	250	0.21	−45.1 ± 1.0
P[EE(**O**)E(H)E(**W**)E(Glc)]	390	340	0.24	350	0.26	−39.8 ± 3.5
P[EE(**O**)E(**F**)E(**H_6_-pept**)]	390	330	0.21	340	0.08	−40.3 ± 0.4

* Calculated by Instrument software; ** Calculated by authors based on SD values provided by Instrument software, as described elsewhere [51].

**Table 3:** Characteristics of the PTX-loaded nanoparticles (50 µg of PTX/mg polymer).

Sample	DLS			ζ-Potential (mV)
	*D_H_* (nm) (by Intensity)	*D_H_* (nm) (by Number)	PDI	
P[EE(**R**)E(H)E(**F**)E(Glc)]	255	−	0.41	−41.9 ± 2.9
P[EE(**O**)E(H)E(**F**)E(Glc)]	200	−	0.35	−39.9 ± 3.9
P[EE(**R**)E(H)E(**I**)E(Glc)]	270	200	0.25	−40.9 ± 1.2
P[EE(**O**)E(H)E(**I**)E(Glc)]	260	190	0.18	−43.8 ± 1.7
P[EE(**R**)E(H)E(**W**)E(Glc)]	170	155	0.42	−36.2 ± 0.7
P[EE(**O**)E(H)E(**W**)E(Glc)]	290	205	0.27	−38.7 ± 0.2
P[EE(**O**)E(**F**)E(**H_6_-pept**)]	335	240	0.21	−33.5 ± 6.0

**Table 4:** Cytostatic effect of the developed PTX nanoformulations (A549 cell line, 72 h).

Sample	IC_50_ (ng/mL)
PTX	0.8 ± 0.1
PTX-LANS	2.0 ± 0.3
PTX/P[EE(**R**)E(H)E(**F**)E(Glc)]	7.8 ± 2.0
PTX/P[EE(**R**)E(H)E(**F**)E(Glc)]	3.6 ± 1.1
PTX/P[EE(**R**)E(H)E(**I**)E(Glc)]	1.3 ± 0.7
PTX/P[EE(**O**)E(H)E(**I**)E(Glc)]	3.0 ± 1.5
PTX/P[EE(**R**)E(H)E(**W**)E(Glc)]	3.7 ± 2.3
PTX/P[EE(**O**)E(H)E(**W**)E(Glc)]	3.9 ± 1.0
PTX/P[EE(**O**)E(**F**)E(**H_6_-pept**)]	7.2 ± 1.1

## Data Availability

The data supporting the findings of this study are available within the article or its Supplementary Materials.

## Supplementary Materials

The following supporting information can be downloaded at: https://www.mdpi.com/article/10.3390/ijms24021049/s1.
