# Supplementary material for: Amphiphilic Polypeptides Obtained by the Post-Polymerization Modification of Poly(Glutamic Acid) and Their Evaluation as Delivery Systems for Hydrophobic Drugs
Source: Int J Mol Sci. 2023 Jan 5;24(2):1049. doi: 10.3390/ijms24021049 (PMC9864831; doi:10.3390/ijms24021049)
Supplement: Supplementary file 1 [file ijms-24-01049-s001.zip › ijms-2034661-supplementary.pdf]

International Journal of *Molecular Sciences*

MDPI

Supplementary Materials

Amphiphilic Polypeptides Obtained by the Post-Polymerization Modification of Poly(Glutamic Acid) and Their Evaluation as Delivery Systems for Hydrophobic Drugs

Apollinariia Yu. Dzhuzha [1,2], Irina I. Tarasenko [2], Leonard Ionut Atanase [3], Antonina Lavrentieva [4] and Evgenia G. Korzhikova-Vlakh [2,*]

1 Institute of Chemistry, Saint-Petersburg State University, 198504 St. Petersburg, Russia;
2 Institute of Macromolecular Compounds, Russian Academy of Sciences, 199004 St. Petersburg, Russia;
3 Faculty of Dental Medicine, "Apollonia" University, 700399 Iasi, Romania;
4 Institute of Technical Chemistry, Gottfried-Wilhelm-Leibniz University, 30167 Hannover, Germany;

Correspondence: vlakh@hq.macro.ru (E.K.-V.)

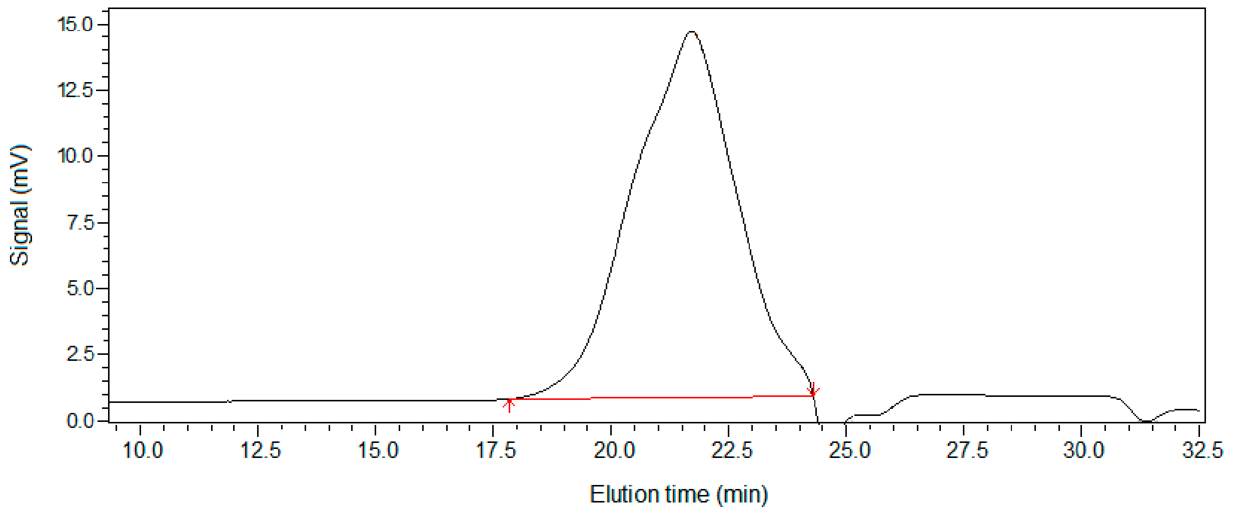

Figure S1. SEC trace of poly(α,L-glutamic acid γ-benzyl ester). Conditions: Styragel Column, HMW6E, Waters (7.8 mm × 300 mm, 15–20 μm bead size), DMF containing 0.1M LiBr, 40 °C, elution rate 0.3 mL/min, refractometric detection.

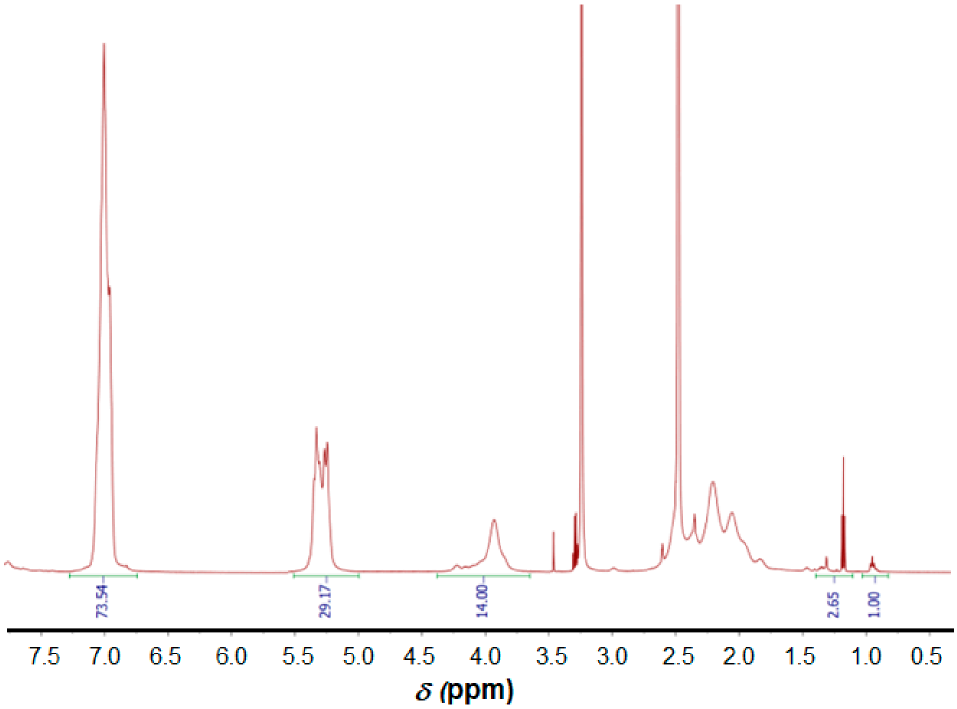

(a)

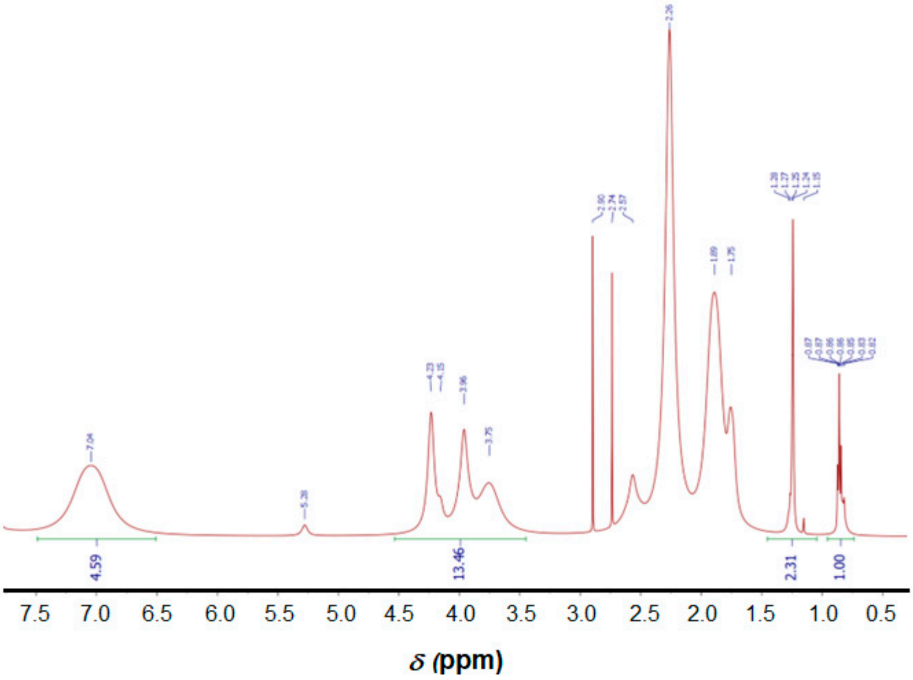

(b)

Figure S2. ^{1}H NMR spectra of Bzl-protected **(a)** and deprotected **(b)** poly(α,L-glutamic acid) (DMSO-d$_6$, 25 °C): *Signals* (ppm): 0.80-0.85 (C<u>H</u>$_3$, hexylamine), 1.00-1.33 (C<u>H</u>$_2$, hexylamine), 3.5-4.5 (C<u>H</u>, Glu), 5.0-5.5 (O-<u>CH$_2$</u>-C$_6$H$_5$, Glu(OBzl)), 6.7-7.4 (O-CH$_2$-<u>C$_6$H$_5$</u>, Glu(OBzl)).

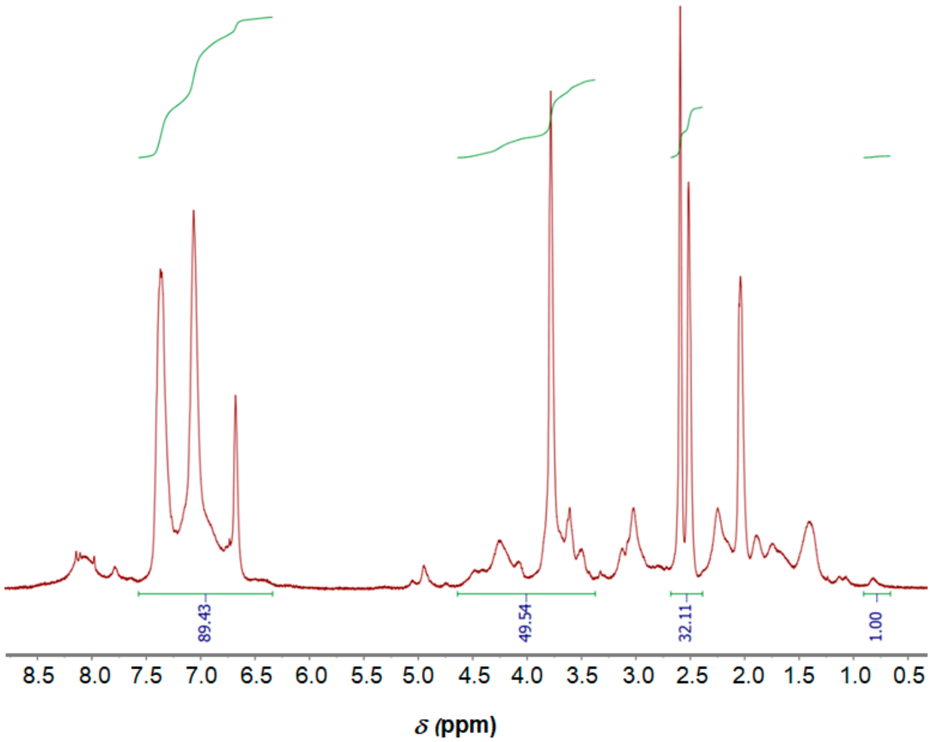
89.43
49.54
32.11
1.00
8.5 8.0 7.5 7.0 6.5 6.0 5.5 5.0 4.5 4.0 3.5 3.0 2.5 2.0 1.5 1.0 0.5
δ (ppm)

(a)

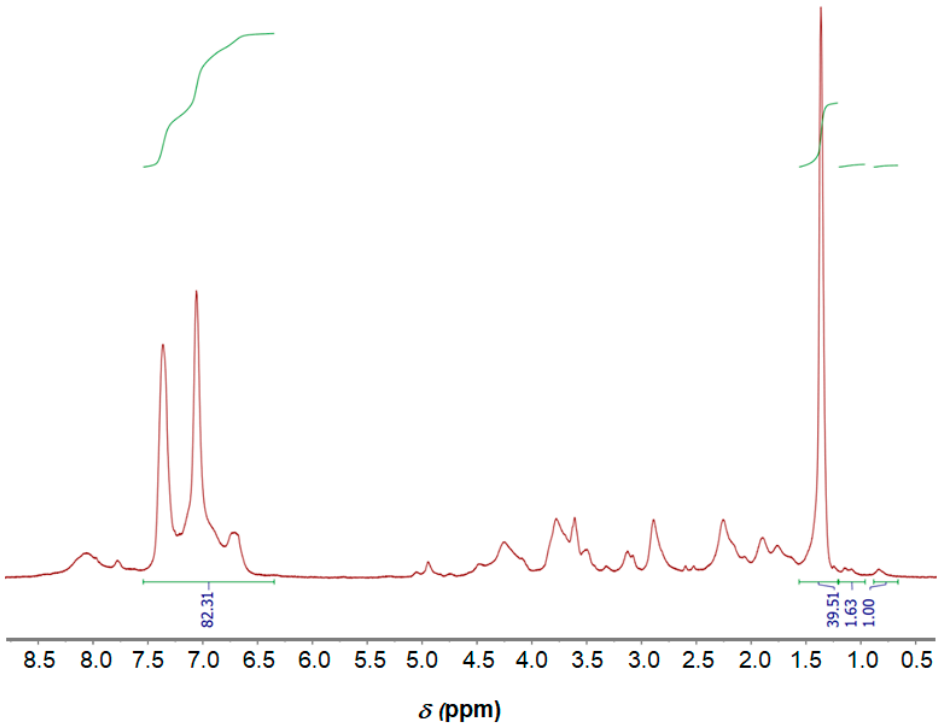
82.31
39.51
1.63
1.00
8.5 8.0 7.5 7.0 6.5 6.0 5.5 5.0 4.5 4.0 3.5 3.0 2.5 2.0 1.5 1.0 0.5
δ (ppm)

(b)

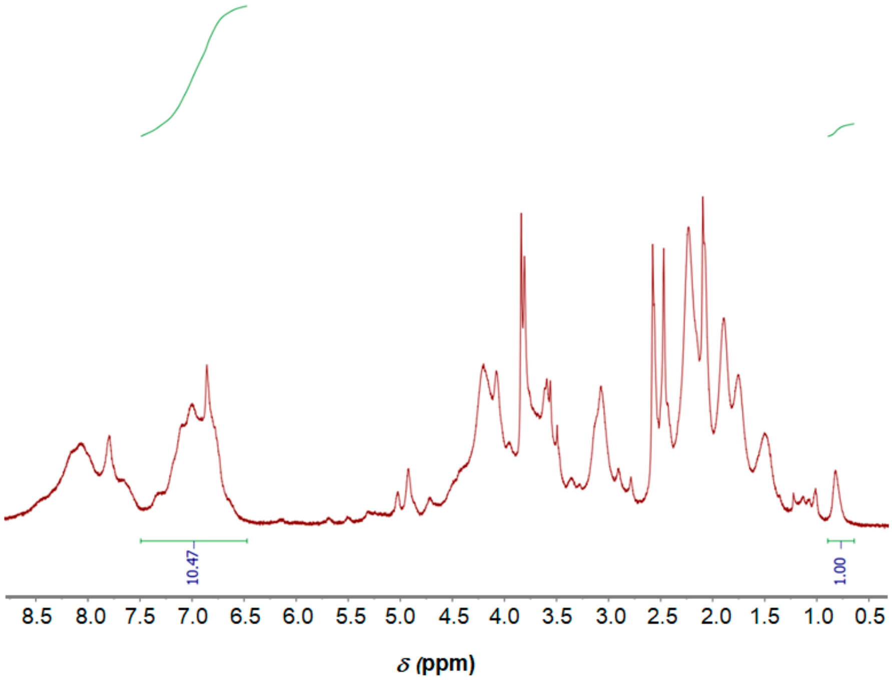

(c)

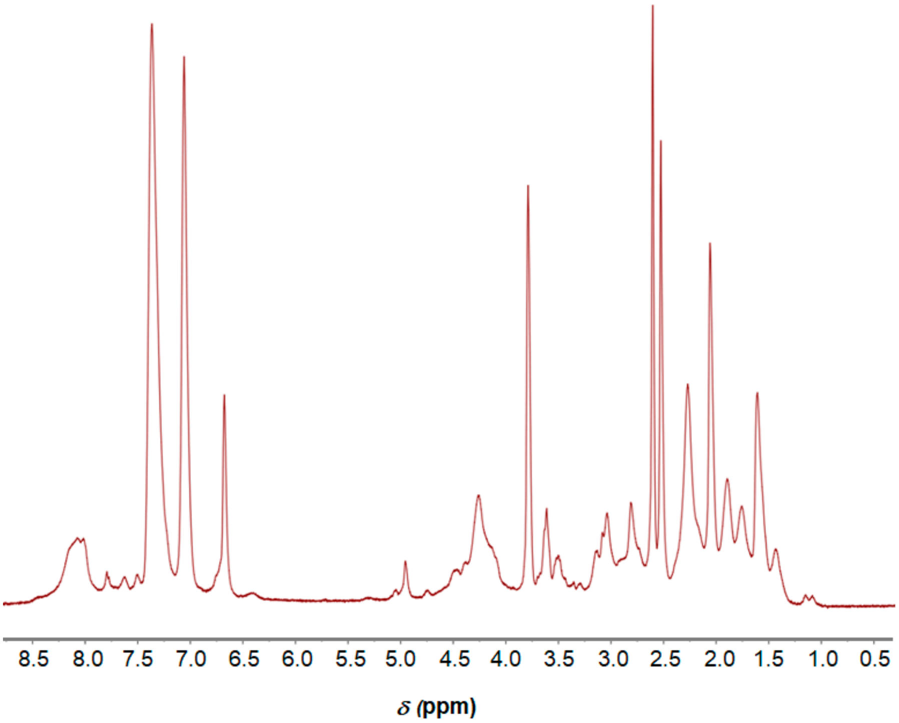

(d)

Figure S3. ^{1}H NMR spectra of P[EE(**R**(Mtr))E(H(Trt))E(**I**)E(Glc)] (a), P[EE(**O**(Boc))E(H(Trt))E(**I**)E(Glc)] (b), P[EE(**R**)E(H)E(**I**)E(Glc)] (with diffusion filter) **(c)**, P[EE(**R**(Mtr))E(H(Trt))E(**W**(Boc))E(Glc)] (with diffusion filter) **(d)**. *Signals* (ppm): 0.80-0.85 ($C\underline{H}_3$, hexylamine), 1.00-1.33 ($C\underline{H}_2$, hexylamine), 1.2-1.5 ($2C\underline{H}_2$, Arg; $C\underline{H}_3$, Boc of Orn and Trp), 1.5-2.4 ($2C\underline{H}_2$, Glu, Arg and Orn), 2.4-2.7 ($C\underline{H}_3$-C_6H_5, Mtr of Arg), 2.7-3.5 (6C$\underline{H}$, glucose; CH_2, Orn), 3.5-4.5 (C$\underline{H}$, Glu; $C\underline{H}_3$-O-C_6H_5, Arg; C$\underline{H}$, Arg, His, Orn, Trp; $C\underline{H}_2$, His), 6.3-7.5 ($C_6\underline{H}_5$, Trt of His; 2$\underline{H}$, His; $C_6\underline{H}_5$, Mtr of Arg; $C_6\underline{H}_5$, Trp), 1.4 ($C\underline{H}_3$, BOC of Orn), 7.8-8.5 (N$\underline{H}$-CO, all polypeptides; $N\underline{H}_2$-C=N$\underline{H}$, Arg).

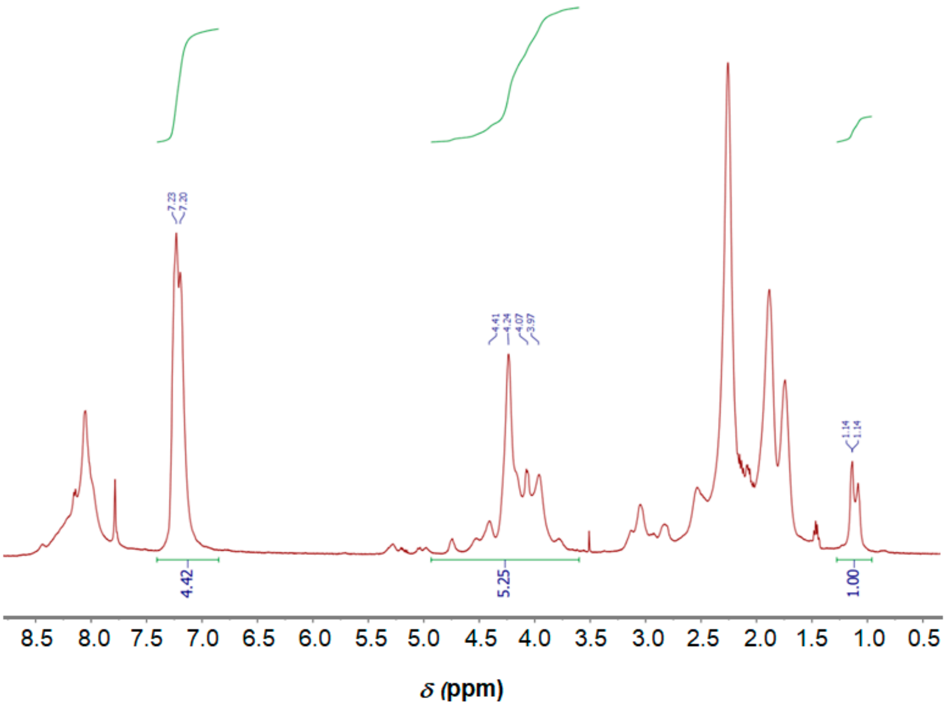
7.23
7.20
4.41
4.24
4.07
3.97
1.14
1.14
4.42
5.25
1.00
8.5 8.0 7.5 7.0 6.5 6.0 5.5 5.0 4.5 4.0 3.5 3.0 2.5 2.0 1.5 1.0 0.5
δ (ppm)

(a)

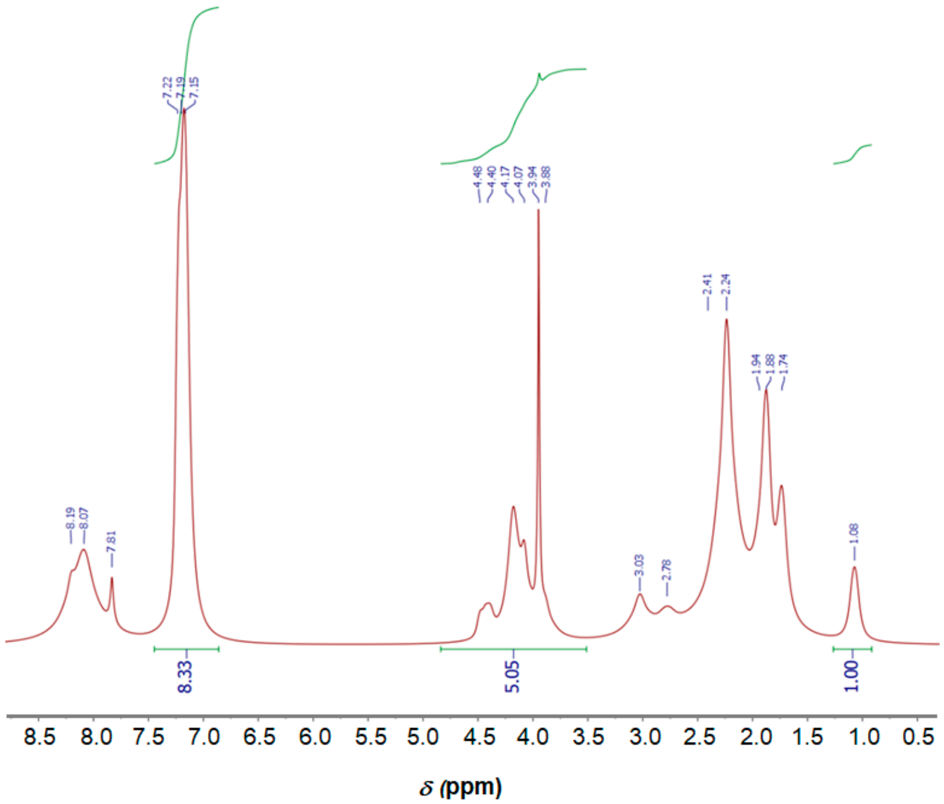
7.22
7.19
7.15
4.48
4.40
4.17
4.07
3.94
3.88
2.41
2.24
1.94
1.88
1.74
8.19
8.07
7.81
3.03
2.78
1.08
8.33
5.05
1.00
8.5 8.0 7.5 7.0 6.5 6.0 5.5 5.0 4.5 4.0 3.5 3.0 2.5 2.0 1.5 1.0 0.5
δ (ppm)

(b)

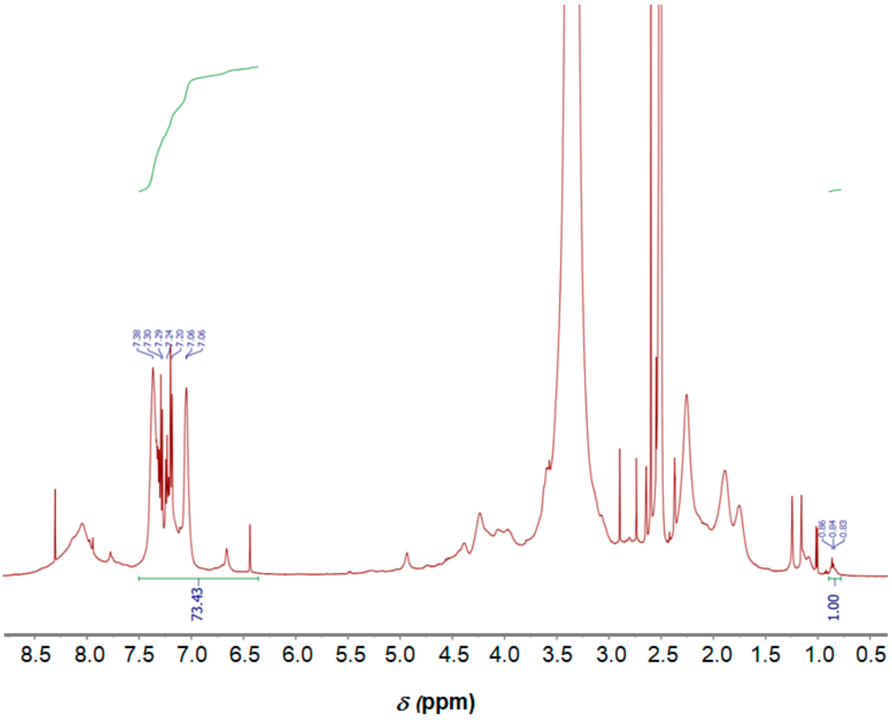

(c)

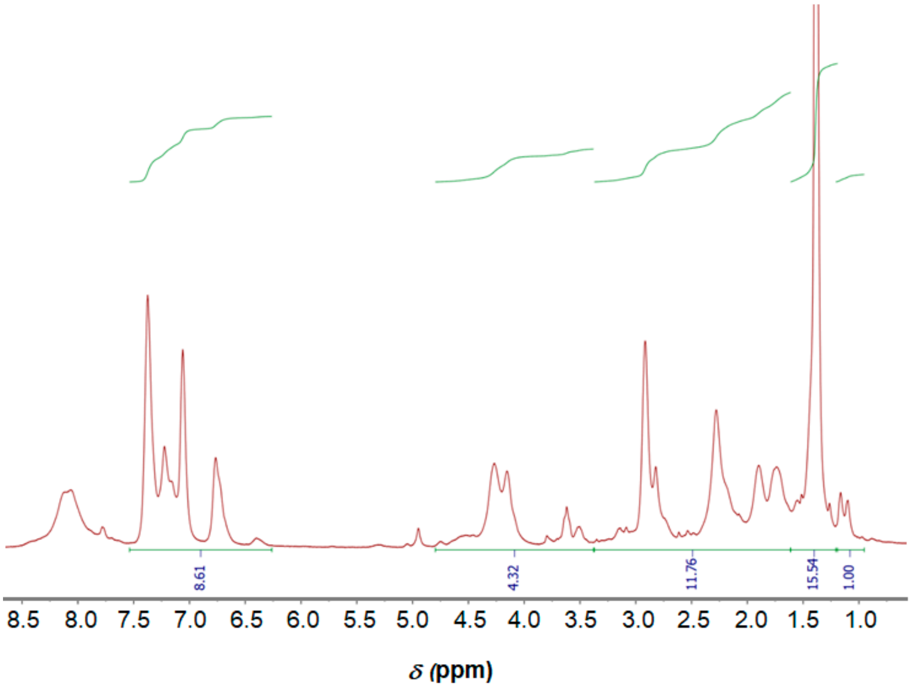

(d)

Figure S4. ^{1}H NMR spectra (DMSO-d_6, 25 °C) of poly(α,L-glutamic acid) modified with: **(a, b)** Phe (a – precursor polymer for P[EE(**O**)E(H)E(**F**)E(Glc)]; b – precursor polymer for P[EE(**O**)E(**F**)E(**H$_6$-pept**)]); **(c)** Phe, His(Trt) and Glc; **(d)** Phe, Orn(Boc), His(Trt) and Glc (with diffusion filter). *Signals* (ppm): 0.80-0.85 (CH_3, hexylamine), 1.00-1.3 (CH_2, hexylamine), 1.3-1.5 (CH, Boc of Orn), 1.5-2.4 (2CH_2, Glu, and Orn), 2.7-3.5 (6CH, glucose; CH_2, Orn), 3.5-4.5 (CH, Glu; CH, His and Orn; CH_2, His), 6.3-7.5 (C_6H_5, Trt of His; 2H, His), 7.8-8.5 (NH-CO, all polypeptides; NH_2-C=NH, Arg).

Table S1. Composition of the amphiphilic copolymers determined by ^{1}H NMR spectroscopy.

Sample	Modifier				
	Determined composition (mol%) *				
	R or *O*	*H*	*Glc*	*F or I*	*H_6-pept.*
P[EE(O)E(H)E(F)E(Glc)]	33	26	17	10	–
P[EE(R)E(H)E(I)E(Glc)]	25	24	21	N/D	–
P[EE(O)E(H)E(I)E(Glc)]	31	24	19	N/D	–
P[EE(O)E(F)E(H_6-pept)]	42	–	–	26	N/D

*Side-chain amino acids and glucosamine were calculated relative to glutamic acid, taken as 100%.

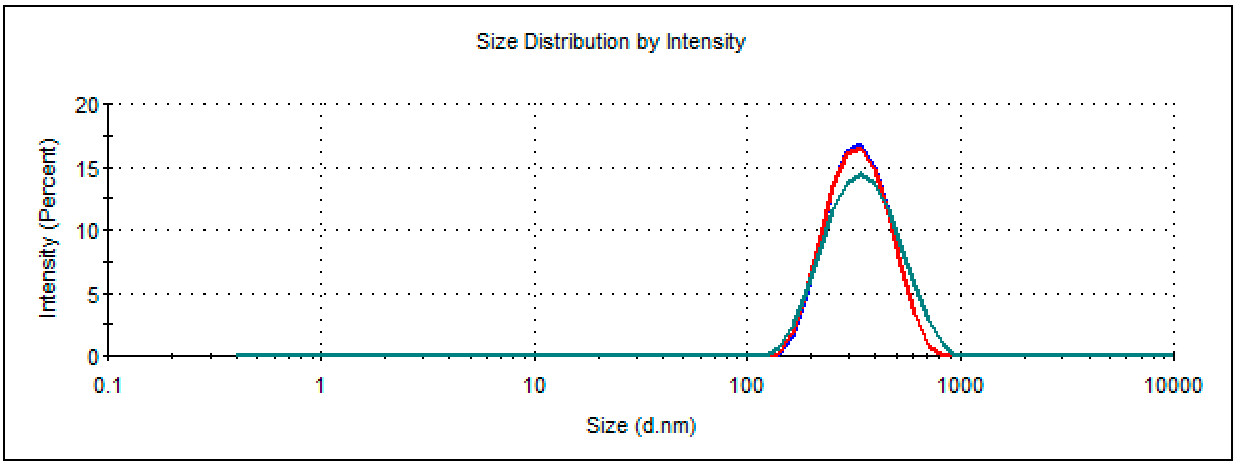

(a)

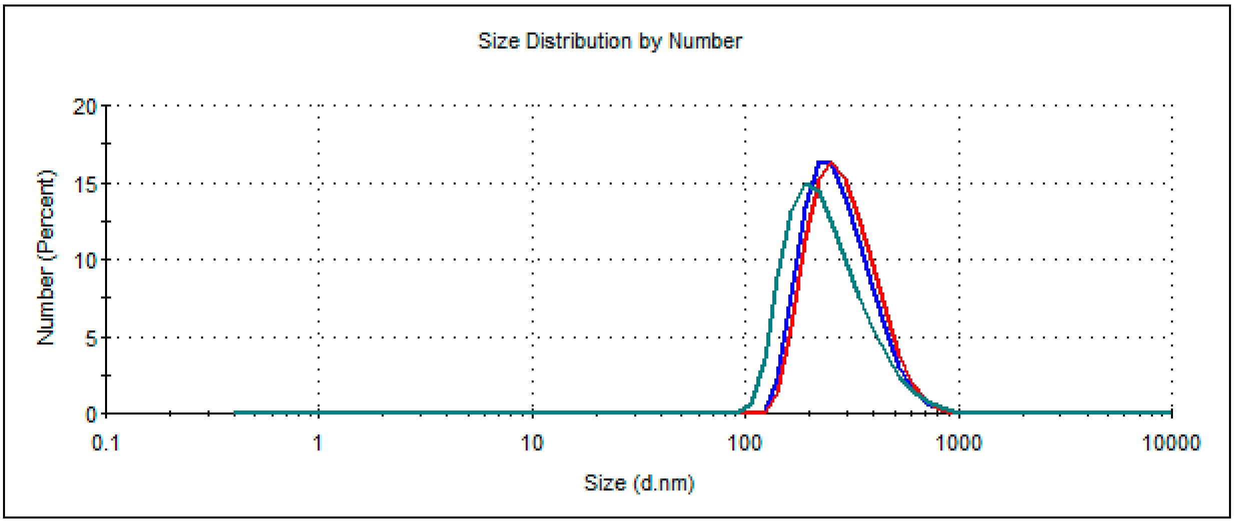

(b)

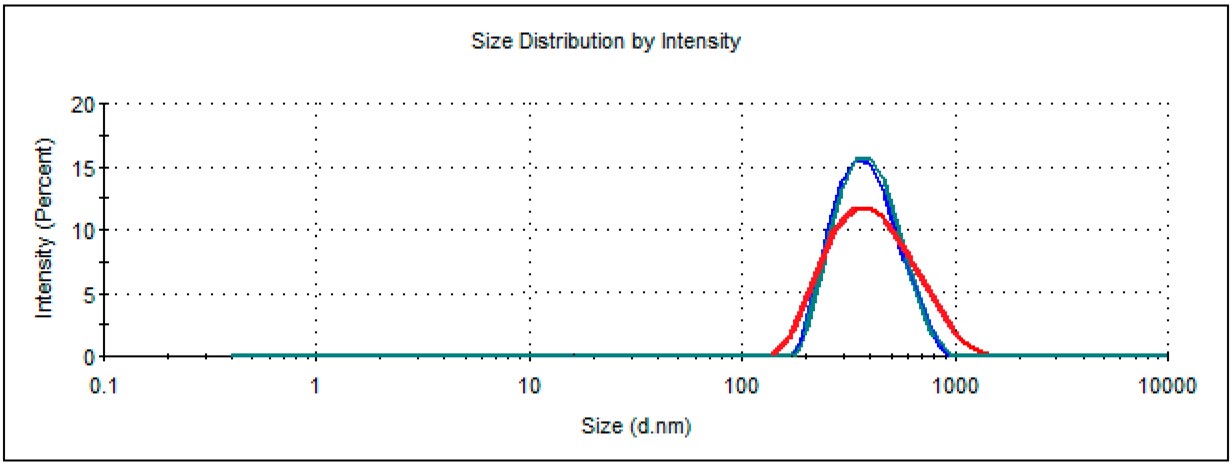

(c)

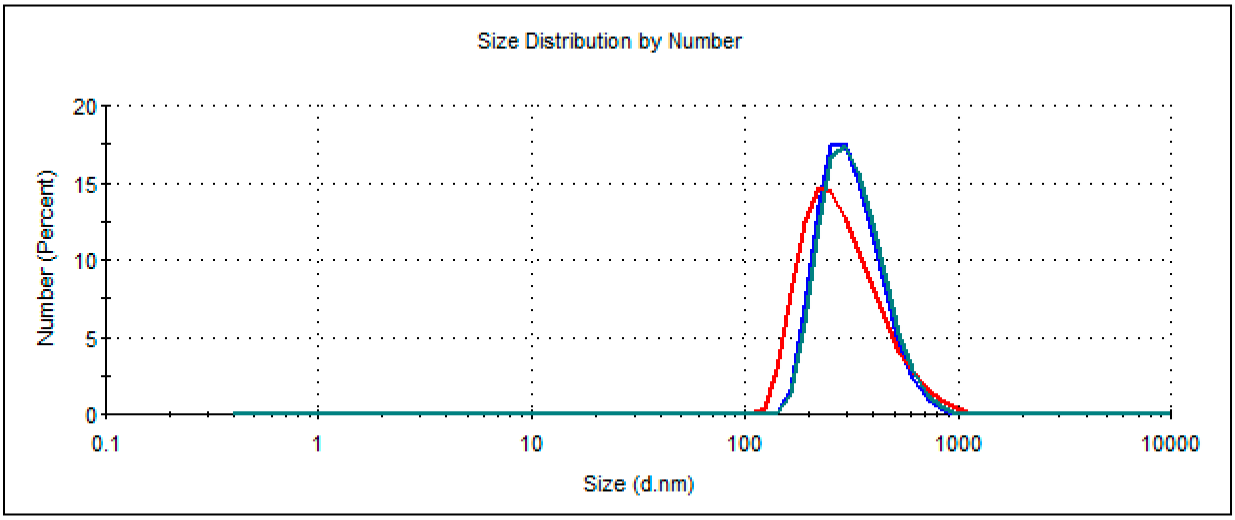

(d)

Figure S5. Hydrodynamic diameter distribution measured by intensity **(a,c)** and by number **(b,d)** (DLS) for P[EE(**O**)E(H)E(**I**)E(Glc)] **(a,b)** and P[EE(**O**)E(**F**)E(**H$_6$-pept**)] **(c, d)** nanoparticles.

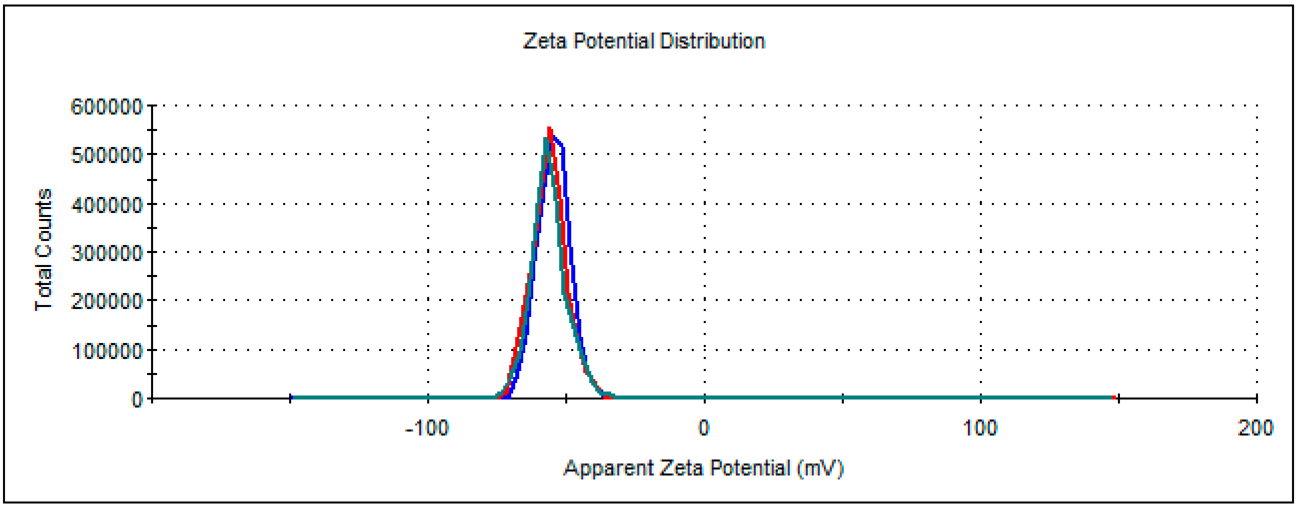

(a)

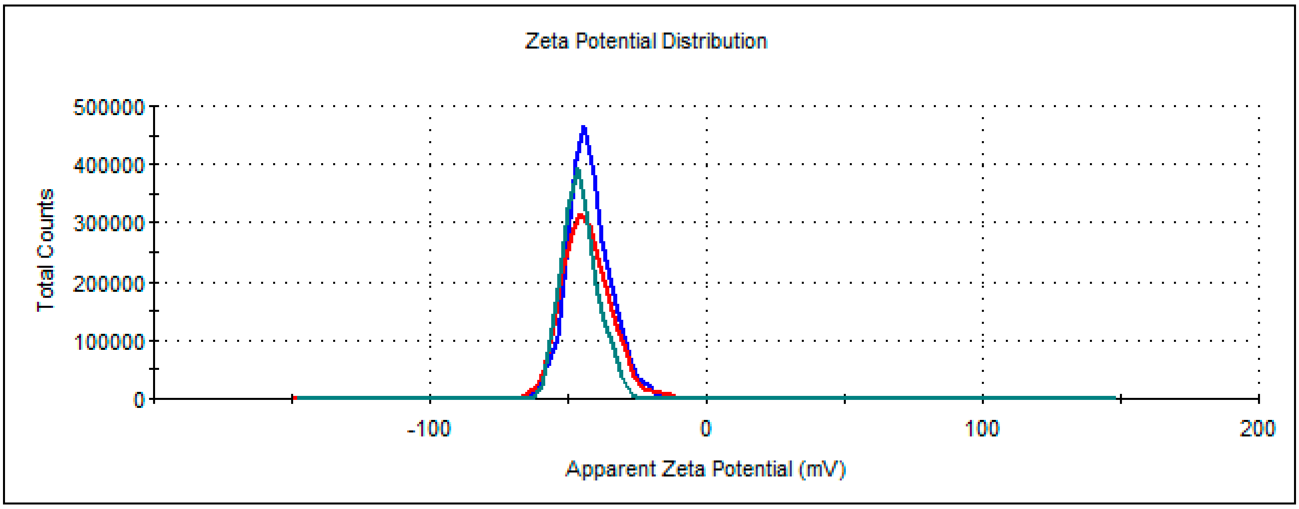

(b)

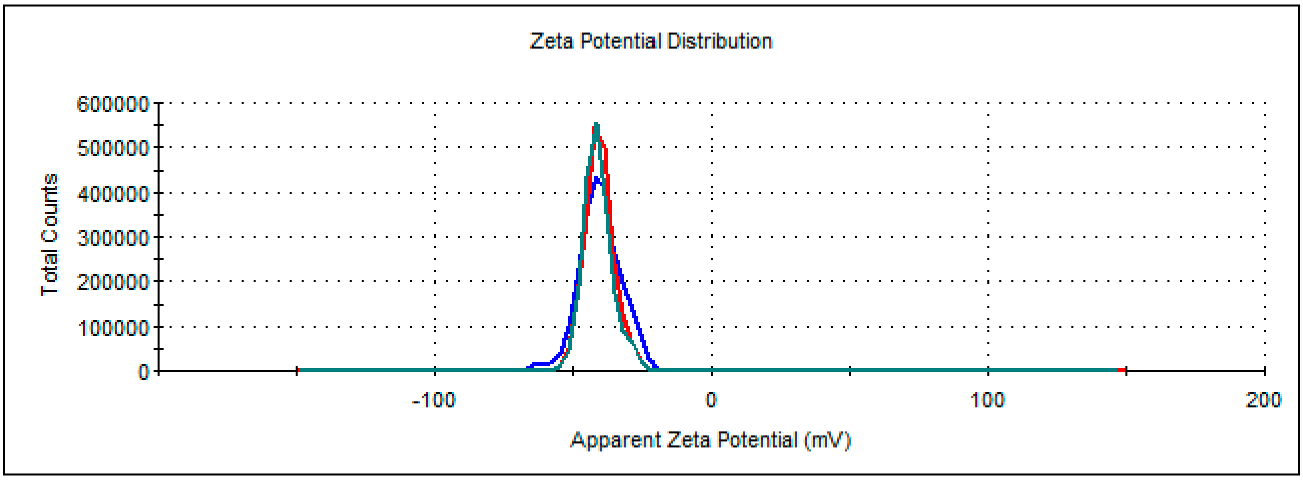

(c)

Figure S6. Zeta-potential distribution obtained by ELS for P[EE(**R**)E(H)E(**I**)E(Glc)] **(a),** P[EE(**O**)E(H)E(**I**)E(Glc)] **(b)** and P[EE(**O**)E(**F**)E(**H_6-pept**)] **(c)** nanoparticles.

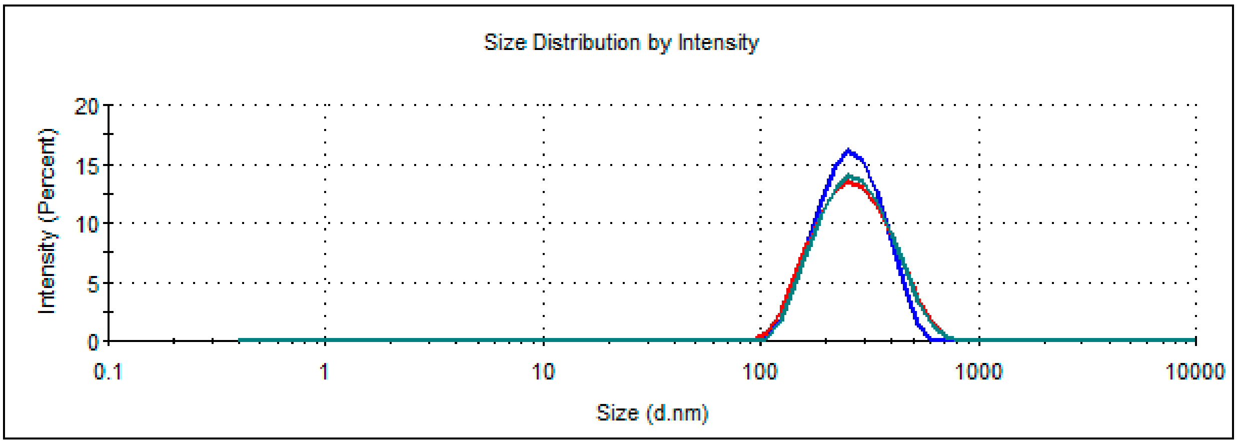
Size Distribution by Intensity
Intensity (Percent)
Size (d.nm)
0
5
10
15
20
0.1
1
10
100
1000
10000

(a)

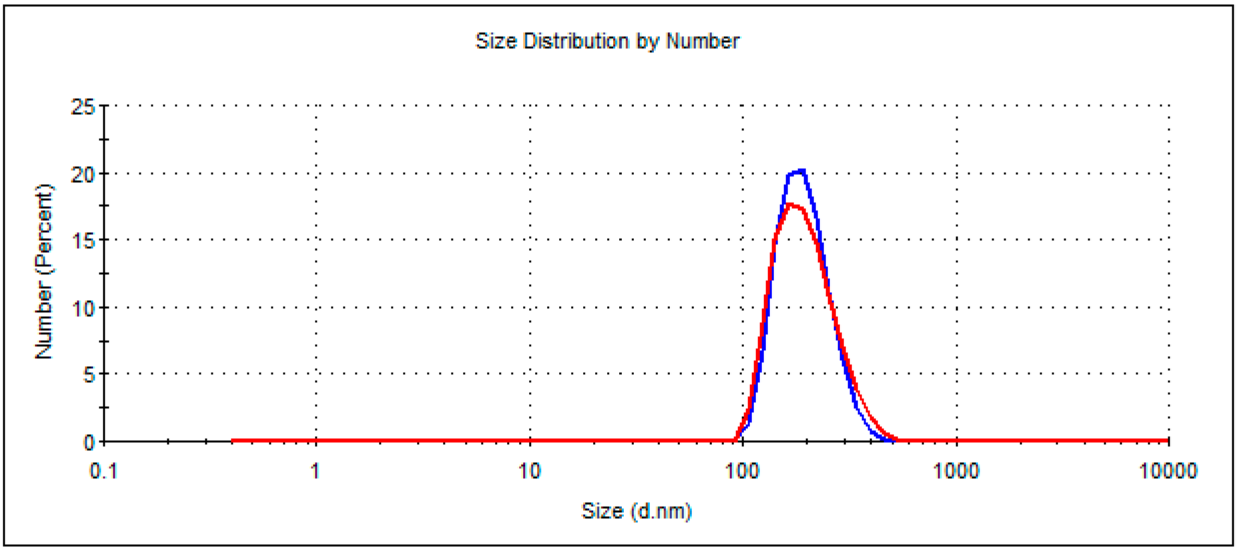
Size Distribution by Number
Number (Percent)
Size (d.nm)
0
5
10
15
20
25
0.1
1
10
100
1000
10000

(b)

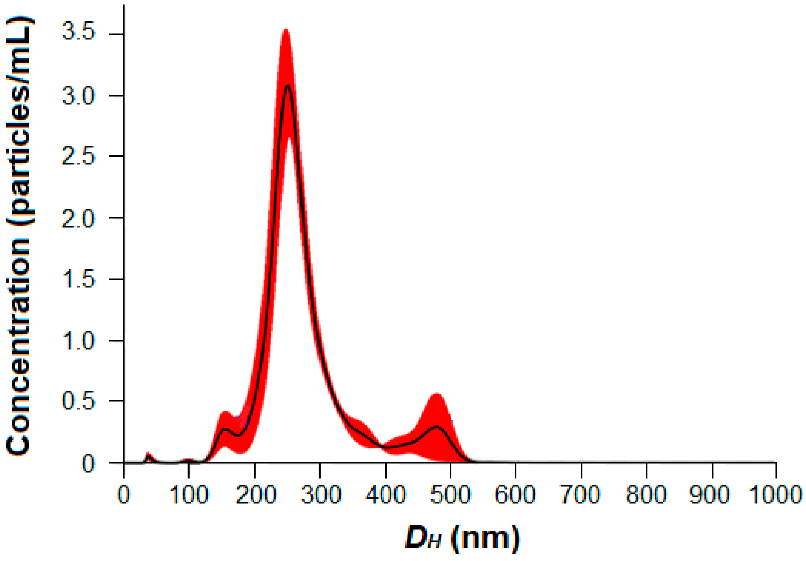
Concentration (particles/mL)
0
0.5
1.0
1.5
2.0
2.5
3.0
3.5
0
100
200
300
400
500
600
700
800
900
1000
DH (nm)

(c)

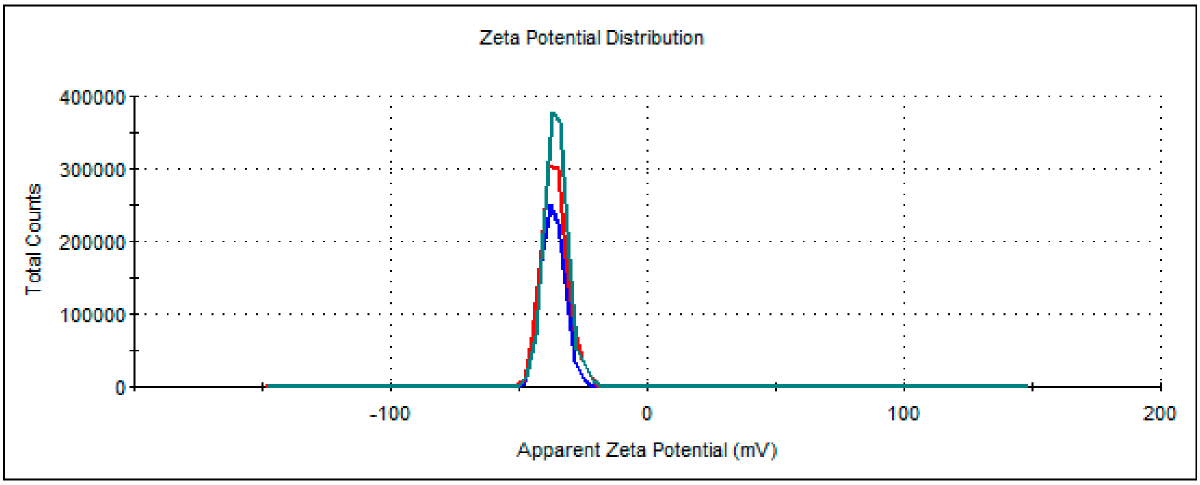

(d)

Figure S7. Hydrodynamic diameter distribution **(a-c)** and zeta-potential distribution **(d)** measured for PTX-loaded nanoparticles based on P[EE(**R**)E(H)E(**I**)E(Glc)] using: DLS by intensity **(a),** DLS by number **(b)**, NTA **(c)**, ELS **(d).**

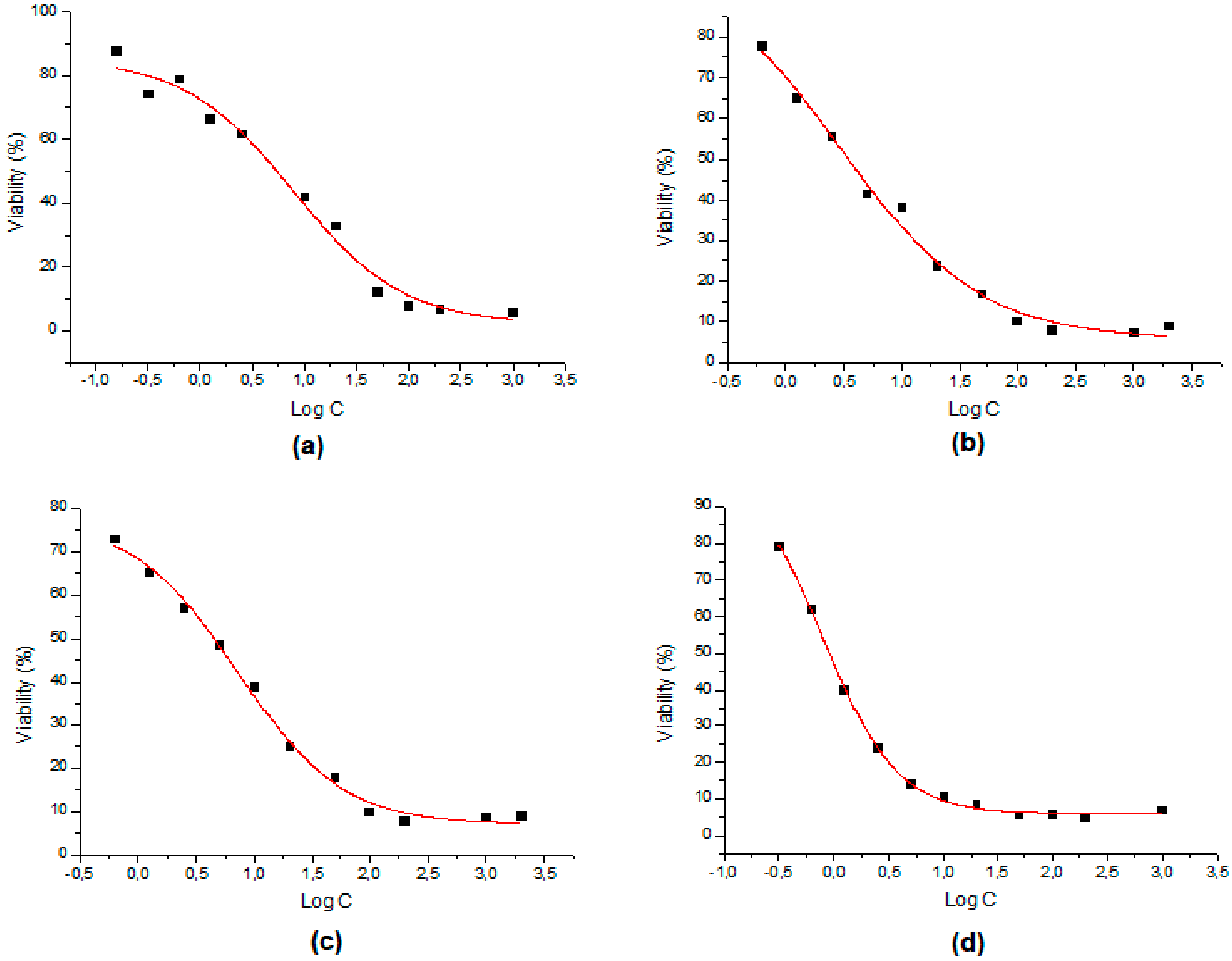

Figure S8. Dose response curves illustrating the inhibition of A549 by PTX nanoformulations **(a-c)** and free PTX **(d)**: PTX/ P[EE(**R**)E(H)E(**F**)E(Glc)] **(a)**, PTX/ P[EE(**O**)E(H)E(**I**)E(Glc)] **(b)**, PTX/ P[EE(**O**)E(**F**)E(**H_6-pept**)] **(c)**.

(a)

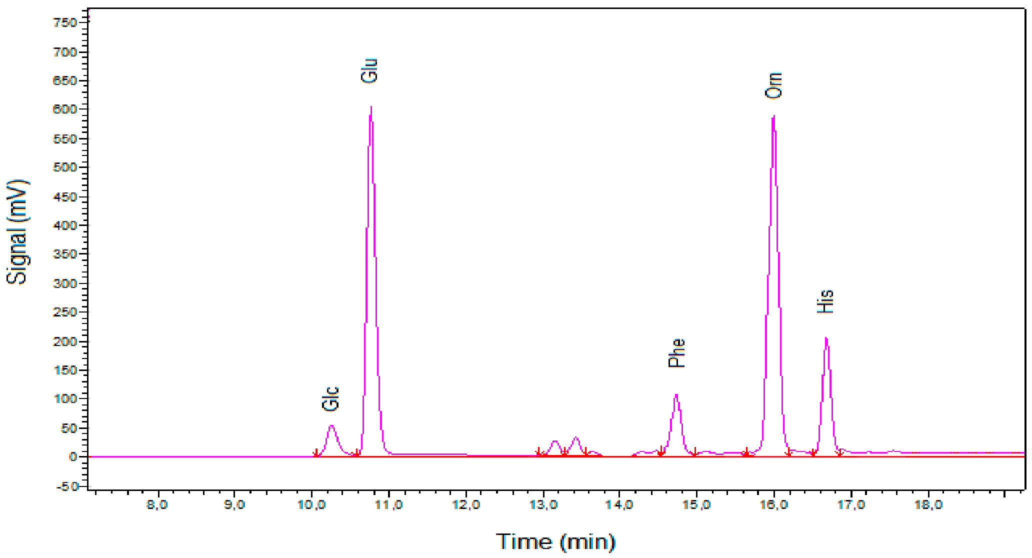
Signal (mV)
Time (min)
Glc
Glu
Phe
Orn
His

(b)

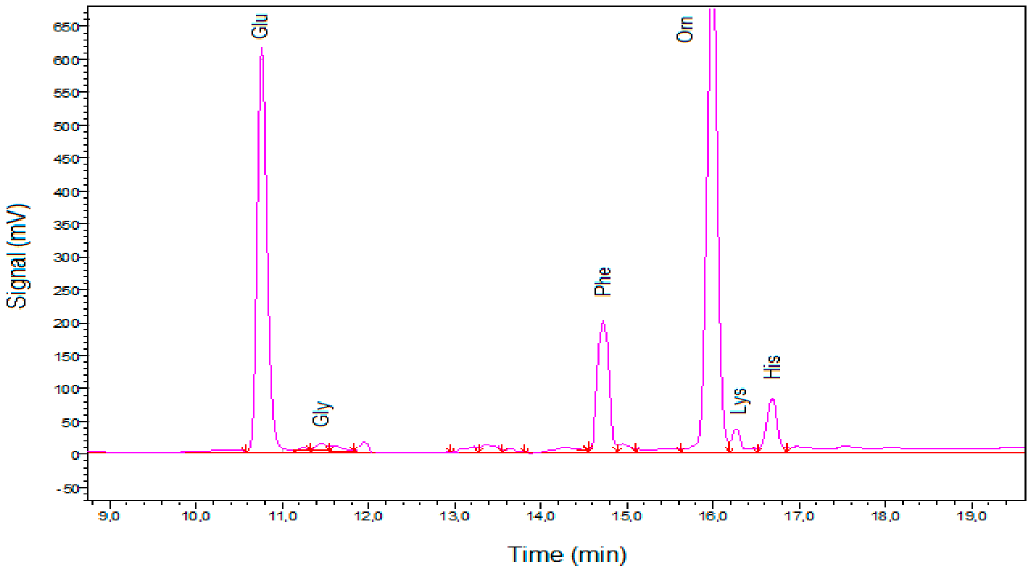
Signal (mV)
Time (min)
Glu
Gly
Phe
Orn
Lys
His

(c)

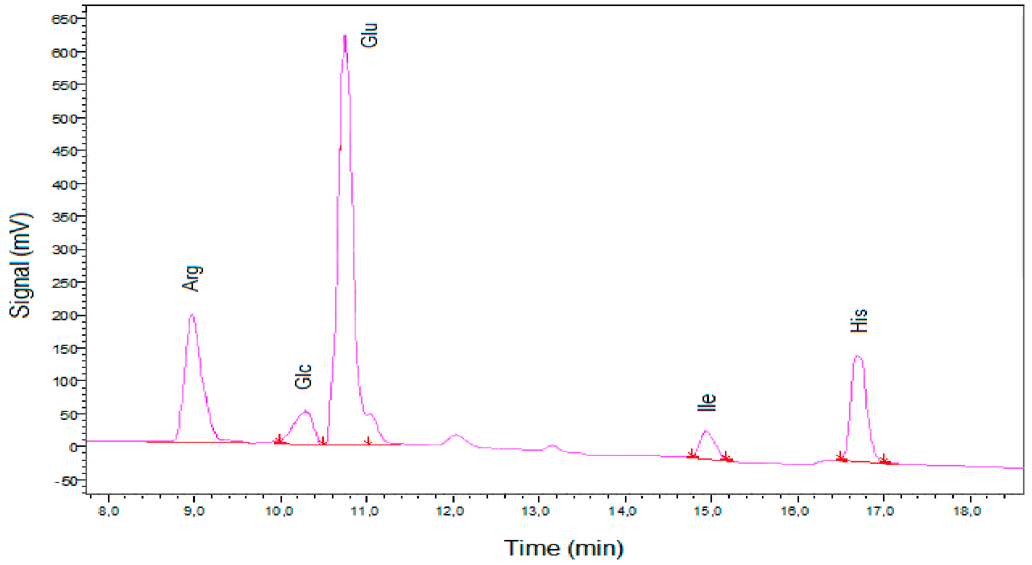
Signal (mV)
Time (min)
Arg
Glc
Glu
Ile
His

(d)

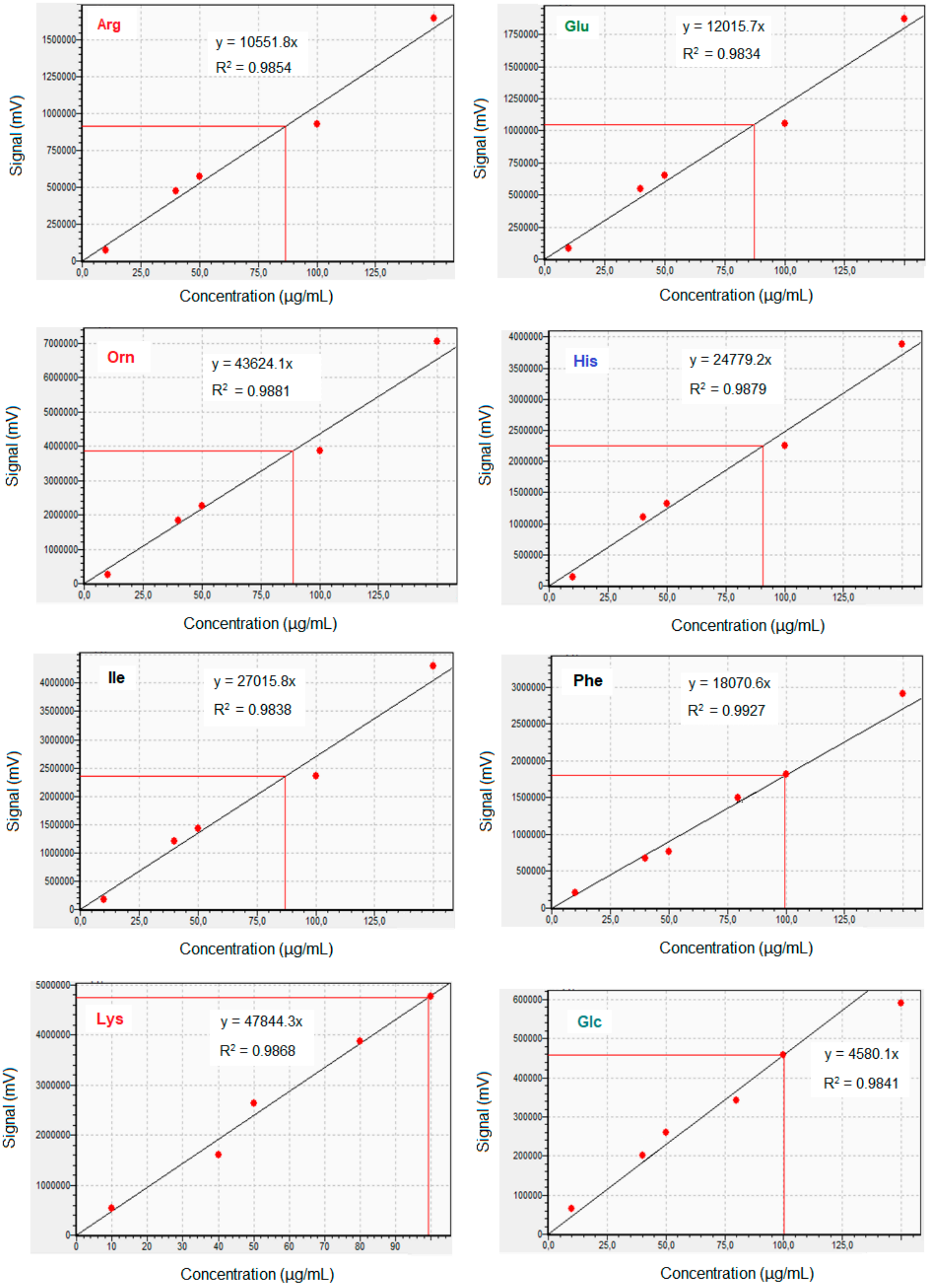

Figure S9. Chromatograms of HPLC amino acid and glucosamine analysis **(a-c)**, obtained after total acidic hydrolysis of (glycol)polypeptides: P[EE(**O**)E(H)E(**F**)E(Glc)] **(a)**, P[EE(**O**)E(**F**)E(**H$_6$-pept**)] **(b)**, P[EE(**R**)E(H)E(**F**)E(Glc)] **(c)** and calibration curves built for individual components **(d)**.
